# Celastrol-Based Hybrid Prodrug Ameliorates ALI by Spatiotemporally Consecutive Dual-Targeting GLUT1/Drp1 to Reestablish Mitochondrial Homeostasis

**DOI:** 10.7150/ijbs.133756

**Published:** 2026-08-21

**Authors:** Hong Xu, Hongyu Chen, Fengling He, Xinlan Li, Wanxin Li, Qingqing Yang, Tingting Zhang, Jin Liu, Xiaoke Shi, Shilin Chen, Defang Li, Jun Lu

**Affiliations:** 1 Chinese Medicine Germplasm Resources Innovation and Effective Uses Key Laboratory of Sichuan Province, Institute of Herbgenomics, School of Pharmacy, Chengdu University of Traditional Chinese Medicine, Chengdu, China;; 2 Featured Laboratory for Biosynthesis and Target Discovery of Active Components of Traditional Chinese Medicine, School of Traditional Chinese Medicine, Binzhou Medical University, Yantai, Shandong, China;; 3 Institute of Herbgenomics, Innovative Institute of Chinese Medicine and Pharmacy, Chengdu University of Traditional Chinese Medicine, Chengdu, China;; 4 Institute for Advancing Translational Medicine in Bone & Joint Diseases, School of Chinese Medicine, Hong Kong Baptist University, Hong Kong SAR.

**Keywords:** Celastrol, M1 macrophage glucose metabolism, Mitochondrial homeostasis, Drp1, Acute lung injury

## Abstract

Aberrant activation of macrophages and their amplification of inflammatory responses constitute the core pathological basis driving the progression of acute lung injury (ALI). Celastrol (CE), despite its potent anti-inflammatory activity, suffers from poor aqueous solubility and substantial systemic toxicity, which severely limit its clinical translation. Capitalizing on the metabolic signature of pro-inflammatory M1 macrophages, specifically their high expression of glucose transporter 1 (GLUT1), we designed a glucose-modified CE prodrug that self-assembled into carrier-free nanoparticles CG NPs. With markedly improved solubility and systemic stability, CG NPs rapidly and persistently accumulated in the inflammatory lungs of LPS-induced ALI mice facilitated by GLUT1-mediated targeting and uptake by M1 macrophages. Compared with free CE, CG NPs exhibited enhanced overall therapeutic efficacy while significantly reducing hepatorenal toxicity. Mechanistic studies revealed that by targeting Drp1, CG NPs disrupt Drp1-MiD51 interaction, thus inhibiting excessive mitochondrial fission and ROS accumulation, which blocks NF-κB-mediated inflammatory signaling and M1-driven cytokine release. Molecular docking suggested that glucose conjugation may confer CG with a superior ability to regulate mitochondrial homeostasis over CE, potentially driven by its unique U-shaped conformation that inserts into Drp1 and forms a denser hydrogen-bond network, which could contribute to enhanced binding affinity. In summary, this study proposes a nanoprodrug strategy that combines precise targeting with mitochondrial protection, offering a promising therapeutic avenue for inflammatory diseases such as ALI.

## Introduction

Acute lung injury (ALI) is a severe and diffuse pulmonary disorder triggered by non-cardiogenic etiologies such as bacterial or viral pneumonia and sepsis. In severe cases, it can progress to acute respiratory distress syndrome, with a mortality rate as high as 30-40% 1-3. The core pathological mechanism involves aberrant macrophage activation and the dysregulated inflammatory cascade they propagate 4, 5. During the acute inflammatory phase, alveolar-resident macrophages are activated predominantly through canonical signaling pathways like NF-κB, polarizing toward a pro-inflammatory M1 phenotype. Subsequently, large amounts of reactive oxygen species (ROS) and inflammatory mediators including TNF-α, IL-1β, and IL-6 are released, leading to diffuse pulmonary edema and impaired gas exchange function 6-8. Notably, M1 macrophages undergo significant metabolic reprogramming during activation, shifting their energy source from oxidative phosphorylation to aerobic glycolysis. Concurrently, glucose transporter 1 (GLUT1) expression is markedly upregulated, providing a molecular basis for targeted delivery strategies 9, 10. Previous studies have demonstrated that glucose-modified nanocarriers can significantly enhance cellular uptake in M1 macrophages via GLUT1-mediated endocytosis, thereby promoting drug accumulation and retention at inflammatory sites.

Mitochondria, as highly dynamic organelles, maintain structural homeostasis through a balance between fusion and fission processes 11-13. Recent evidence indicates that excessive mitochondrial fission plays a pivotal regulatory role in macrophage inflammatory activation. Fragmented mitochondrial networks not only impair oxidative phosphorylation capacity but also lead to sustained accumulation of mitochondrial reactive oxygen species (mtROS), thereby triggering the NF-κB pathway and NLRP3 inflammasome, intensifying the inflammatory cascade 14-16. Dynamin-related protein 1 (Drp1), a key GTPase mediating mitochondrial fission, serves as a central molecular link between cellular stress and mitochondrial morphological remodeling 17, 18. Under inflammatory stimuli, oxidative stress, or pathogen-associated molecular pattern activation, multiple signaling pathways promote phosphorylation of serine 616 (S616) in Drp1. Consequently, Drp1 translocates to the outer mitochondrial membrane, where it binds to receptor proteins such as Fis1, MFF, and MiD49/51, ultimately leading to excessive mitochondrial fragmentation. Substantial evidence indicates a strong positive correlation between abnormal Drp1 activation and the intensity of macrophage inflammatory responses in models of lipopolysaccharide (LPS) stimulation, bacterial infection, and sterile inflammation 19, 20. Moreover, in experimental models of ALI and sepsis, attenuating Drp1 activity or disrupting its mitochondrial translocation effectively ameliorates mitochondrial function and mitigates tissue injury.

Celastrol (CE), a triterpenoid natural product, exhibits broad-spectrum anti-inflammatory, antioxidant, and immunomodulatory activities 21-23. Its mechanisms involve inhibiting key inflammatory signaling pathways such as NF-κB and STAT3, downregulating the expression of pro-inflammatory cytokines including TNF-α, IL-1β, and IL-6, and thereby mitigating excessive pulmonary inflammation 24, 25. Furthermore, CE modulates macrophage polarization by suppressing the M1 phenotype and ameliorates the inflammatory microenvironment through mitochondrial protection and oxidative stress reduction. However, its clinical translation is significantly hampered by poor water solubility, lack of targeting specificity, low bioavailability, and potential systemic toxicity.

Drug conjugation technology involves the covalent linkage of a targeting ligand to a potent therapeutic agent via a chemical linker, enabling selective drug enrichment at the disease site. This strategy not only reduces off-target toxicity but also improves the physicochemical and pharmacokinetic properties of the drug 13, 26-29. Successful implementation of this strategy hinges on three critical factors: stable linkers ensuring circulatory integrity; highly efficient targeted ligands enabling specific recognition and internalization by diseased cells; and rational design simultaneously enhancing solubility, stability, and biocompatibility. Glucose (Glu), an endogenous small molecule, offers excellent biosafety and a polyhydroxy structure amenable to chemical modification, effectively improving the hydrophilicity of conjugates 30, 31. Given that M1 macrophages highly express GLUT1 and exhibit enhanced Glu uptake under inflammatory conditions, this study designed and constructed a Glu-conjugated CE prodrug which self-assembled into nanoparticles CG NPs, using CE as the therapeutic payload and Glu as the targeting ligand. The objective is to enhance drug accumulation in M1 macrophages through GLUT1-mediated targeted delivery and to investigate the mechanism by which this nanosystem modulates mitochondrial homeostasis to suppress inflammatory responses, thereby providing a novel strategy for the precise ALI treatment.

## Materials and methods

### Chemicals and reagents

Celastrol (CE) was purchased from Adamas (Chengdu, China); β-D-glucose pentaacetate, boron trifluoride etherate, Fmoc-Gly-ol, octanedioic acid, and rhodamine B were obtained from Macklin (Shanghai, China). Piperidine, potassium hydroxide, N,N,N′,N′-tetramethyl-O-(7-azabenzotriazol-1-yl)uronium hexafluorophosphate (HATU), N,N-diisopropylethylamine (DIPEA), and 4-dimethylaminopyridine (DMAP) were all supplied by Rhawn (Shanghai, China). Other solvents and reagents were acquired from Jinshan Chemical Reagent (Chengdu, China), all of analytical grade and used as received. Reaction progress was monitored by thin-layer chromatography (TLC) and visualized under UV light at 254 nm. All compounds were purified by flash column chromatography on silica gel (200-300 mesh). The ^1^H and ^13^C NMR spectra were recorded on a Bruker spectrometer (AVANCE NEO 600 MHz). Chemical shifts (*δ*) are reported in ppm using tetramethylsilane (TMS) as an internal standard. Residual solvent peaks were used as internal references (CDCl_3_: *δ* 7.26 ppm for ^1^H, *δ* 77.2 ppm for ^13^C; CD_3_OD-*d*_4_: *δ* 3.31 ppm for ^1^H, *δ* 49.0 ppm for ^13^C, DMSO-*d*_6_: *δ* 2.50 ppm for ^1^H, *δ* 39.5 ppm for ^13^C). The signals were described as singlet (s), doublet (d), triplet (t), double doublet (dd), double triplet (dt), triple doublet (td), quartet doublet (qd), broad signal (br) and multiplet (m). *J* values (coupling constants) were expressed in hertz (Hz). Accurate mass measurements were performed on the electrospray ionization (ESI) apparatus using an Agilent 1260-Bruker tims TOF mass. IR spectra were recorded in the spectral range of 4000~400 cm^-1^ using a Nicolet iN10 FTIR spectrometer (Thermo Scientific).

DMEM medium was purchased from Corning (New York, USA). Fetal bovine serum (FBS) and bovine serum albumin (BSA) were supplied by ExCell Bio (Shanghai, China). Penicillin-streptomycin and trypsin were provided by Biofrox (Cambridge, UK). Antibodies against p65 (#WL01980), p-p65 (#WL02169), IKK (#WL00053), p-IKK (#WLA0347), IκB (#WL01936), p-IκB (#WL02495), GLUT1 (#WL01163), Mfn1 (#WL06394), Mfn2 (#WL06347), GAPDH (#WL01114), β-actin (#WL01372) were obtained from Wanleibio (Shenyang, China). ARG1 (#ab96183) and iNOS (#ab178945) monoclonal antibodies were obtained from Abcam (Cambridge, MA, USA). Anti-MFF antibody (#HA723630) was purchased from Hangzhou Huaan Biotechnology (Hangzhou, China). p-DRP1 (Ser616) antibody (#AWA10727) was obtained from Abiowell Biotechnology (Changsha, China). Antibodies against MiD51 (#20164-1-AP), Drp1 (#12957-1-AP) and Fis1 (#10956-1-AP) were obtained from Proteintech (Wuhan, China). AffiniPure Goat Anti-Mouse IgG (H+L), Mouse IL-1β ELISA Kit, Mouse tumor necrosis factor-α (TNF-α) ELISA Kit, and Mouse IL-6 ELISA Kit were bought from Boster (Wuhan, China). Interleukin-4 (IL-4) and Interleukin-13 (IL-13) were purchased from Chamot Biotechnology Co., Ltd. (Shanghai, China). The DAPI Staining Solution, Triton X-100, methylthiazolyldiphenyl-tetrazolium bromide (MTT), LDH Cytotoxicity Assay Kit, Crystal violet, Reactive Oxygen Species (ROS) Assay Kit, Mitochondrial Superoxide Assay Kit with MitoSO™ Red, Mitochondrial membrane potential assay kit with JC-1, ATP Assay Kit, Radio Immunoprecipitation Assay Lysis buffer (RIPA), Phenylmethanesulfonyl fluoride (PMSF) solution, Phosphatase inhibitor cocktail, Hematoxylin and Eosin (H&E) Staining Kit, AF647-labeled Goat Anti-Rabbit IgG (H+L) antibody, AF488-labeled Goat Anti-Rabbit IgG (H+L), Nitric Oxide (NO) Assay Kit, and ECL Imaging Reagent were provided by Beyotime (Shanghai, China). Lipopolysaccharide (LPS) and Protein A/G Immunoprecipitation Kit were bought from Biosharp (Hefei, China). PKMito Orange dye was obtained from Genvivo Biotech (Nanjing, China). MitoTracker Deep Red FM was obtained from Thermo Fisher Scientific (MA, USA). Evans Blue and Carrageenan were purchased from Aladdin (Shanghai, China). Polyvinylidene fluoride (PVDF) membranes were obtained from Millipore (MA, USA).

### Preparation and characterization of CG NPs

CG NPs were prepared via a standard one-step nanoprecipitation procedure by dropwise addition of a CG solution (8 mg in 600 μL DMSO) into 5 mL of ultrapure water under vigorous stirring at room temperature, followed by continuous agitation for 2 h. After 24 h of dialysis to remove the organic solvent, yellow CG NPs were obtained by freeze-drying.

The hydrodynamic size, polydispersity index (PDI), and zeta potential of CG NPs were measured by dynamic light scattering (Litesizer DLS 500, Anton Paar, Austria). Morphology was observed by transmission electron microscopy (TEM, Hitachi HT-7800, Japan). The Tyndall effect was confirmed by red laser irradiation. The solubility changes were observed by dissolving equimolar amounts of CE, glucose (Glu), and CG NPs in distilled water. The oil-water partition coefficient logP values were predicted using the online tool SwissADME (http://www.swissadme.ch).

### Stability test

1 mg of CG NPs was dissolved in 2.5 mL of PBS (pH = 7.4) to form a 0.4 mg/mL solution, and stored at 4 ℃. The particle size stability was analyzed using DLS after 0, 1, 3, 5, 7, 9, 11, 13, and 15 days of storage.

1 mg of CG NPs was added to 2.5 mL of PBS (pH = 7.4) containing 10% fetal bovine serum (FBS) or 10% rat plasma/heparin to form 0.4 mg/mL solutions. The samples were incubated at 37 ℃ for 0, 8, 16, 24, 48, and 72 h to evaluate serum stability. At each designated time point, samples were collected and analyzed for particle size changes using DLS.

### Molecular dynamics simulation and molecular docking

The molecular dynamics simulation was initiated by geometry optimization at the HF/6-31G level using Gaussian 16 software, where the self-consistent field calculation was conducted to locate the minimum-energy conformation, with atomic charges derived through the Merz-Kollman method. Subsequently, a simulation system containing 30 CG molecules in an 8 nm cubic box was constructed using GROMACS 2020.7 with the GAFF2 force field and SPC/E water model. After energy minimization via the conjugate gradient algorithm, the system underwent sequential equilibration for 0.1 ns under NPT and NVT ensembles. Production molecular dynamics simulation was then performed for 100 ns under NPT conditions at 300 K and 1 bar, employing a 1.0 nm cutoff for non-bonded interactions, the Particle Mesh Ewald method for long-range electrostatics, and the Lennard-Jones potential for van der Waals interactions. Configurations were extracted at specific timepoints throughout the trajectory, with subsequent visualization and analysis conducted using VMD 1.9.3 and GROMACS 2020.7 respectively. Molecular docking was performed using Schrödinger Glide standard precision. The Drp1 protein structure (PDB ID: 8T1H) was obtained from the Protein Data Bank (http://www.rcsb.org/).

### Cells and animals

RAW 264.7, THLE-2, AML-12, HEK293, and HK-2 cells were purchased from Wuhan Pricella Biotechnology Co., Ltd. Cells were cultured in DMEM high-glucose medium supplemented with 10% FBS and 1% penicillin-streptomycin solution, in a 5% CO_2_, 37 ℃ constant temperature incubator. Cells in the logarithmic growth phase were used for the experiments.

Healthy male BALB/c mice (6-8 weeks old) were provided by Chengdu Shudaqiang Biotechnology Co., Ltd (Chengdu, China). All animal procedures were approved by the Experimental Animals Administrative Committee of Chengdu University of Traditional Chinese Medicine (No. 2026021) and were performed according to the relevant animal regulations.

### Viability and cytotoxicity assays

Cells were seeded in 96-well plates. Following cell adhesion, cells were exposed to a series of drug concentrations for 24 h. MTT solution (0.5 mg/mL final concentration) was then added and incubated for 2 h at 37 ℃. After careful removal of the supernatant, DMSO was added to dissolve the formazan crystals. Absorbance was measured at 490 nm using a microplate reader (FlexStation3, Molecular Devices). Cytotoxicity was evaluated by measuring lactate dehydrogenase (LDH) release. After drug treatment, 120 μL of cell culture supernatant was transferred to a 96-well plate, mixed with 60 μL LDH detection working solution, and incubated for 30 min in the dark at room temperature. Absorbance was measured at 490 nm. Cytotoxicity was calculated as:



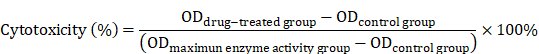



### Induction and validation of macrophage polarization

To induce polarization, RAW 264.7 cells were stimulated with LPS (1 μg/mL) for 24 h to generate M1 phenotype, or with IL-4 and IL-13 (both 20 ng/mL) for 24 h to generate M2 phenotype. Polarization was confirmed by Western blot analysis of the marker proteins iNOS (M1) and Arg1 (M2).

### Western blot

Cells were seeded in 6-well plates, grown to 80% confluency, and treated as indicated. After treatment, cells were lysed on ice with RIPA buffer for 10 min. Lysates were centrifuged and supernatants were collected for protein quantification using a BCA assay. Protein samples were mixed with 5× SDS loading buffer, denatured by boiling at 100 ℃ for 5 min, separated by 10% SDS-PAGE gels, and transferred onto PVDF membranes. Membranes were blocked with 5% skim milk for 2 h at room temperature, incubated with appropriate primary antibodies at 4 ℃ overnight, followed by HRP-conjugated secondary antibodies for 1 h at room temperature. Protein bands were visualized by chemiluminescence using an automated imaging system (OI 600, BIO-OI).

### Immunofluorescence

For immunofluorescence, RAW 264.7 cells were seeded on glass coverslips in 12-well plates (5 × 10^4^ cells/well). After adherence and treatment, cells were fixed with 4% paraformaldehyde for 15 min, permeabilized with 0.3% Triton X-100 in PBS for 15 min, and blocked with 5% BSA for 1 h. Cells were then incubated with primary antibodies (1:200 dilution) overnight at 4 ℃, followed by Alexa Fluor 647-conjugated secondary antibodies for 1 h at room temperature. Nuclei were stained with DAPI, and coverslips were mounted with anti-fade medium. Images were acquired using a laser scanning confocal microscope (LSCM, FV4000, Olympus).

For flow cytometric analysis of GLUT1 expression, RAW 264.7 cells were seeded in 6-well plates (1 × 10^4^ cells/mL), cultured for 24 h, and stained following the above immunofluorescence procedure (without mounting). Cells were then collected and analyzed by flow cytometry (BD FACSVerse, BD Biosciences).

### Cellular uptake and competitive assay

For cellular uptake, RAW 264.7 cells were plated in 6-well plates (1×10^6^ cells/well). After adhesion, cells were treated for 24 h with LPS (1 μg/mL) or IL-4/IL-13 (both 20 ng/mL), then incubated with 1 μM Rhb-CE or Rhb-CG for 2 h. Cells were collected and fluorescence intensity was analyzed by flow cytometry. For the competitive uptake assay, M1 macrophages were pretreated with glucose (60 μM) for 30 min, followed by incubation with 1 μM Rhb-CE or Rhb-CG for 2 h. Cells were subsequently collected and the cellular uptake was quantified by flow cytometry.

### Inflammatory mediators and morphological Analysis

RAW 264.7 cells were seeded in 6-well plates (1×10^6^ cells/well) and treated for 24 h with complete medium (control), LPS (1 μg/mL; model), or LPS plus 1 μM of the corresponding drug (treatment). Cells were then fixed with 4% paraformaldehyde (15 min), stained with 0.5% crystal violet (10 min), and then observed and imaged under an inverted microscope to record morphological changes. Nitric oxide (NO) levels in cell culture supernatants were determined using the Griess method. Intracellular ROS levels were measured using the fluorescent probe DCFH-DA. Cells were seeded in confocal dishes or 6-well plates (5×10^4^ cells/well). After treatment, the medium was replaced with serum-free medium containing 10 μM DCFH-DA and incubated for 30 min in the dark. Cells were then washed with PBS and immediately imaged by LSCM or harvested for flow cytometric analysis. Cytokine levels in lung tissue homogenates or cell culture supernatants were measured by ELISA according to the manufacturer's instructions. Absorbance was read at 450 nm, and concentrations were determined from a standard curve.

### Macrophage polarization detection

After drug treatment, macrophages were collected and washed with PBS. To block Fc receptors, the cells were incubated with purified anti-mouse CD16/CD32 antibody on ice for 15 min. The cells were then stained with FITC-conjugated anti-mouse CD86 antibody on ice for 30 min. After washing with PBS, the cells were fixed with 4% paraformaldehyde for 15 min, followed by permeabilization with 1% Triton X-100 at room temperature for 10 min. The cells were then washed again with PBS and incubated with PE-conjugated anti-mouse CD206 antibody at room temperature for 30 min. Finally, macrophage polarization was analyzed by flow cytometry.

### Mitochondrial function and morphology analysis

MtROS was detected using MitoSOX™ Red. RAW 264.7 cells were seeded in confocal dishes (5 × 10^4^ cells/well), treated as indicated, stained with 5 μM MitoSOX™ Red working solution for 30 min at 37 ℃ in the dark, washed twice with PBS, and imaged by LSCM. ATP levels were measured with the BeyoTime Biotechnology ATP assay kit. After treatment, cells were lysed with pre-cooled RIPA buffer on ice for 10 min. The supernatant was collected by centrifugation, protein concentration was determined by BCA assay, and ATP content was measured according to the kit instructions. Mitochondrial membrane potential (MMP) was assessed using the Enhanced Mitochondrial Membrane Potential Assay Kit (JC-1). Cells seeded in confocal dishes (5×10^4^ cells/well) were treated, washed twice with PBS, incubated with 1 mL JC-1 working solution for 20 min at 37 ℃ in the dark, washed again, and imaged by LSCM.

Mitochondrial morphology was visualized using PKMito Orange. RAW 264.7 cells seeded in glass-bottom confocal dishes were treated, incubated with serum-free medium containing PKMito Orange (1:5000) for 30 min at 37 ℃ in the dark, washed twice with PBS, and imaged using a structured illumination microscopy system (Multi-SIM X, NanoInsights-Tech) with a 63×1.40 M27 Plan-Apochromat Oil Objective (ZEISS) and an sCMOS camera (Kinetix, Teledyne Imaging).

### Animal model establishment and treatment

Mice were anesthetized and placed in a supine position. ALI was induced by slow intratracheal instillation of LPS (5 mg/kg) using a microsyringe; sham control mice received an equal volume of 0.9% NaCl. After instillation, gentle thoracic pressure was applied to distribute the liquid evenly, and the incision was sutured. At 4 h post-LPS, mice were divided into treatment groups and administered CE (1 mg/kg), Glu (0.39 mg/kg), or CG (1.46 mg/kg) via tail vein injection once daily for three consecutive days. Control and model groups received an equal volume of vehicle.

### Pharmacokinetic study

Healthy male BALB/c mice were used for the pharmacokinetic study. Following intravenous administration via the tail vein, approximately 50 μL of blood was collected from the retro-orbital venous plexus at 1, 5, 15, and 30 min, as well as 1, 2, 4, 8, and 12 h post-dose, and transferred into heparinized tubes. Blood samples were centrifuged at 3500 rpm for 10 min at 4 ℃ to separate the plasma. A 20 μL aliquot of plasma was mixed with five volumes of methanol for protein precipitation, vortexed thoroughly, and centrifuged at 13,000 rpm for 10 min. The supernatant was then collected and transferred to another tube, gently dried under a stream of nitrogen at 30 ℃, and reconstituted in methanol. The resulting samples were analyzed by HPLC. Pharmacokinetic parameters were calculated using DAS 2.0 software.

### *In vivo* biodistribution

ALI model mice were injected via tail vein with Rhb-CE (2.5 mg/kg) or Rhb-CG NPs (3 mg/kg). *In vivo* fluorescence imaging was performed under isoflurane anesthesia at 1, 2, 4, and 6 h post-injection using a small-animal imaging system (ABL X6, Tanon; excitation 520 nm, emission 580 nm). After imaging, mice were euthanized and lungs were collected for *ex vivo* fluorescence quantification.

### Respiratory function

Respiratory function was assessed 24 h after the final administration using a non-invasive whole-body plethysmography system (UPWARDS-WBP, DSI) over a 5-minute recording period. Parameters recorded included Enhanced Pause (Penh), minute ventilation (MVb), peak expiratory flow (PEF), and expiratory flow at 50% of tidal volume (EF50).

### Lung tissue analysis

For Evans blue extravasation, Evans blue solution (20 mg/kg) was injected via tail vein and circulated for 1 h. Mice were euthanized, and the pulmonary vasculature was perfused with 30 mL PBS via the left ventricle. Lungs were excised, weighed, homogenized in formamide (1:9, w/v), incubated at 60 ℃ for 24 h, and centrifuged. Supernatant absorbance was measured at 620 nm, and dye content was calculated from a standard curve. For lung wet/dry weight ratio, freshly excised lungs were blotted, weighed (wet weight, W), dried at 80 ℃ for 72 h to constant weight (dry weight, D), and the W/D ratio was calculated. Cytokine levels in lung tissue homogenates or cell culture supernatants were measured by ELISA according to the manufacturer's instructions. Absorbance was read at 450 nm, and concentrations were determined from a standard curve. For histology, lung tissues were fixed in 4% paraformaldehyde for 5 days, processed through dehydration, clearing, paraffin infiltration, and embedding. Sections were stained with hematoxylin and eosin (H&E) for microscopic evaluation.

### *In vivo* Safety Evaluation

Healthy male BALB/c mice (6-8 weeks old) were housed under standard conditions (12 h light/dark, 22 ± 2 ℃, 50 ± 10% humidity) with free access to food and water. After acclimatization, toxicity was evaluated in both acute and long-term studies. For the acute toxicity study, mice were randomized by body weight into nine groups (n = 6 per group): vehicle control (equal volume of solvent), CE groups (0.5, 1, 2, and 4 mg/kg), and CG NPs groups (0.75, 1.5, 3, and 6 mg/kg). CG NPs doses were calculated according to the molecular-weight ratio between CE and CG to achieve equimolar CE-equivalent dosing. All treatments were administered via tail-vein injection once daily for 3 consecutive days. General behavior, body weight changes, and mortality were continuously monitored during dosing and after the final administration. At the end of the study, samples were collected for routine hematological analysis as well as serum biochemical analyses and histopathological evaluation of major organs.

For the long-term toxicity study, mice were randomly assigned (based on body weight) into four groups: control (equal volume of solvent), CE (1 mg/kg), glucose (Glu, 0.39 mg/kg), and CG NPs (1.46 mg/kg). Drugs were administered via tail-vein injection once daily for 30 consecutive days, and body weight was recorded daily. After the final administration, whole blood was collected by retro-orbital sampling, and major organs (heart, liver, spleen, lungs, and kidneys) were harvested for subsequent serum biochemical assays and histopathological examination.

### Hematoxylin and eosin (H&E) staining

Tissue samples from mice were fixed in 4% paraformaldehyde for 5 days, processed through dehydration, clearing, paraffin infiltration, and embedding. Sections were stained with H&E for microscopic evaluation. Images were acquired using a digital slide scanner (S60, Hamamatsu Photonics).

### Hematological and serum biochemical analyses

After the experiment, whole blood was collected from mice under anesthesia via the retro-orbital venous plexus. For routine hematological analysis, blood was collected into anticoagulant tubes and analyzed using a veterinary automated hematology analyzer (BC-2800Vet, Mindray Bio-Medical Electronics) to determine white blood cell count (WBC), monocyte count (Mono), and neutrophil count (Gran), following the manufacturer's instructions. For serum biochemical analysis, whole blood was transferred into 1.5 mL centrifuge tubes, allowed to clot at room temperature for 30 min, and centrifuged at 3,000 × g for 15 min at 4 ℃. The serum supernatant was collected and analyzed using an automated biochemistry analyzer (Chemray240, Rayto) to measure liver function markers alanine aminotransferase (ALT) and aspartate aminotransferase (AST), as well as renal function markers uric acid (UA) and creatinine (CREA).

### Immunofluorescence colocalization analysis

RAW 264.7 cells were seeded on glass coverslips and treated with the indicated agents for 24 h. Mitochondria were labeled in live cells with MitoTracker Deep Red FM (100 nM, 20 min), followed by fixation, permeabilization, and blocking. Cells were then incubated with a rabbit anti-Drp1 primary antibody overnight at 4 ℃ and subsequently with an Alexa Fluor 488-conjugated secondary antibody for 1 h at room temperature in the dark. After mounting, Drp1-mitochondria colocalization was imaged using a laser scanning confocal microscope.

### Cellular Thermal Shift Assay

After treatment with 1 μM CE or CG NPs for 4 h, cells were collected using PBS containing protease inhibitors and transferred to 1.5 mL tubes. The cell suspensions were subjected to a temperature gradient (41 ℃ to 61 ℃) for 3 min, followed by three cycles of rapid freezing in liquid nitrogen and thawing in a 25 ℃ water bath to lyse the cells. The lysates were centrifuged at 12,000 × g for 15 min at 4 ℃. Supernatants were boiled in loading buffer for 5 min and analyzed by Western blotting.

### Co-immunoprecipitation assay

After drug treatment, cells were lysed on ice in pre-chilled lysis buffer containing protease inhibitors, and total protein lysates were collected after centrifugation. Protein A/G magnetic beads were pre-incubated with the specific antibody or isotype IgG for 30 min at room temperature, then incubated with lysates overnight at 4 ℃ with rotation. The beads were washed with cold lysis buffer, proteins were eluted by boiling in 1× SDS sample buffer, and the target protein and its interacting partners were analyzed by Western blotting.

### Data analysis

All data are expressed as mean ± standard deviation (SD) from at least three independent experiments. For comparisons between two groups, an unpaired two-tailed Student's t-test was used. For comparisons among multiple groups, one-way analysis of variance (ANOVA) was performed followed by Bonferroni's post hoc test. The statistical analyses were performed on the GraphPad Prism (version 9.0).

## Results and discussion

### Preparation and characterization of CG NPs

**Figure 1:**
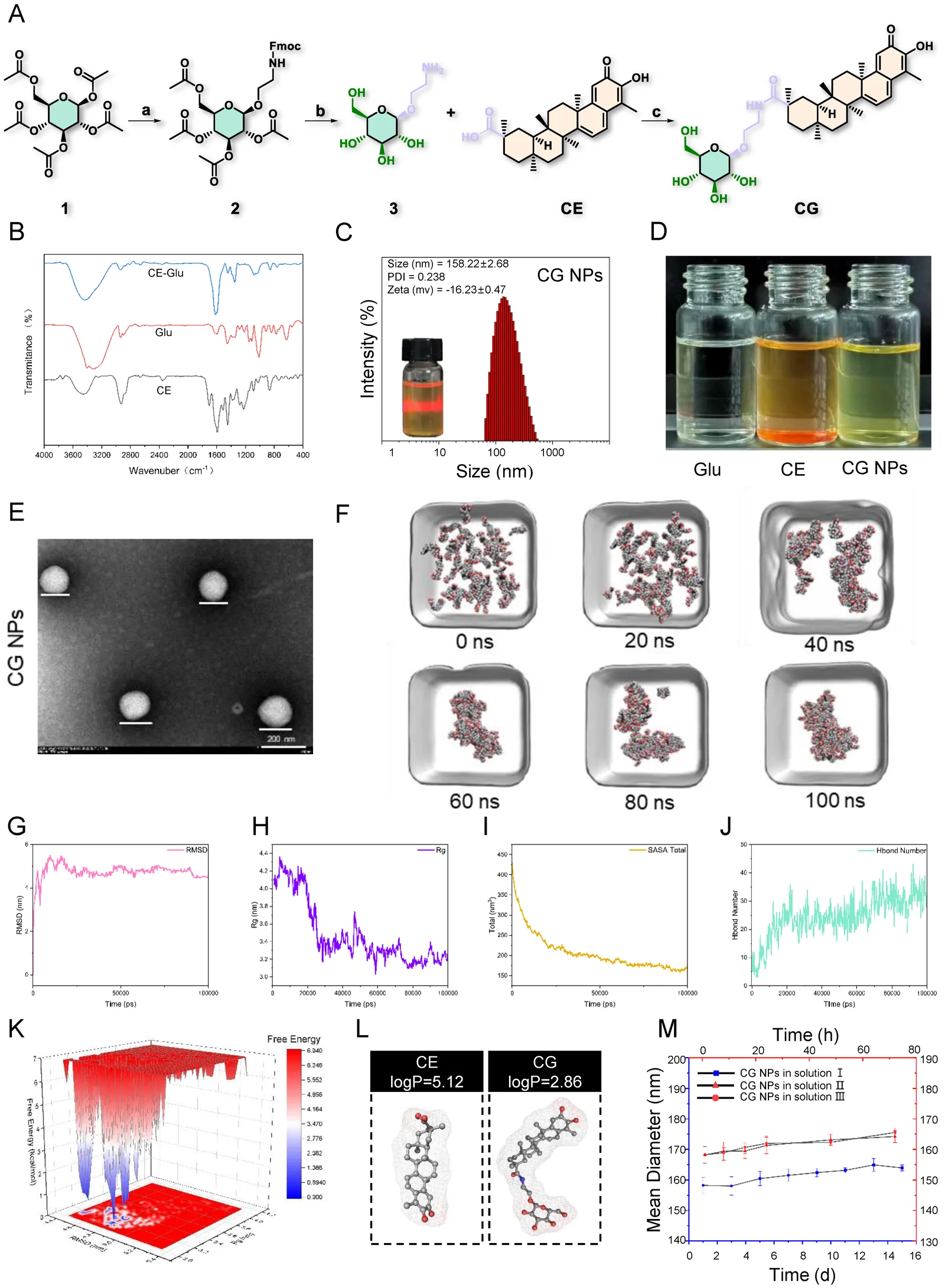
** Physicochemical profiles of CG NPs.** (**A**) Synthesis of CG dimer prodrug. (**B**) The IR spectrogram of Glu, CE and CG. (**C**) The particle size, uniformity, zeta potential, and Tyndall effect of CG NPs (in ultrapure water) (*n* = 3). (**D**) Solubility test of Glu, CE and CG NPs in aqueous solution. (**E**) Representative TEM image of CG NPs. (**F**) Molecular cluster changes of CG during 100 ns simulation (red balls: carbon; blue balls: nitrogen; black balls: oxygen; white balls: hydrogen). (**G**) RMSD diagram of CG NPs during the self-assembly. (**H**) Rg Profile of CG NPs during self-assembly. (**I**) Time-dependent changes in SASA during CG NPs self-assembly. (**J**) Hydrogen bond formation during the self-assembly of CG NPs. (**K**) Free energy landscape of CG NPs. (**L**) The predicted logP values of CE and CG. (**M**) Size changes of CG NPs in PBS (pH = 7.4) (I), PBS (pH = 7.4) containing 10% FBS (II), and PBS (pH = 7.4) containing 10% rat plasma/heparin (III).

Despite its potent anti-inflammatory activity, the clinical translation of CE is hindered by poor aqueous solubility and systemic toxicity 32. To overcome these constraints, we designed a Glu-modified nanoprodrug, CG NPs, aiming to enhance the solubility of CE while enabling active delivery to inflammatory sites. In this construct, Glu was covalently conjugated to CE as a hydrophilic modifier and a targeting ligand (**Figure 1A**). The CG heterodimer was synthesized according to **Scheme S1**, and its structure was confirmed by ¹H NMR and ^13^C NMR spectroscopy, with its purity determined by HPLC (**Figures S1**-**S4**). A rhodamine B-labeled derivative (Rhb-CG) was also prepared for subsequent *in vivo* tracking (**Schemes S2-S4**, **Figures S5-S10**). FT-IR analysis further verified the successful conjugation of CE and Glu, displaying characteristic absorptions of both CE and the Glu moiety, including the C-O-C stretching vibration at 1076.1 cm^-1^ and the aliphatic C-H stretching vibration at 2927.4 cm^-1^. In addition, the emergence of an amide I band at 1639.1 cm^-1^ provided further evidence for amide bond formation between CE and Glu (**Figure 1B**). The amphiphilic CG conjugate readily self-assembled into well-defined nanoparticles CG NPs via nanoprecipitation, exhibiting a distinct Tyndall effect. DLS revealed an average hydrodynamic diameter of 158.22 nm and a zeta potential of -16.23 mV in ultrapure water. The incorporation of Glu significantly improved aqueous dispersibility, resulting in a stable nano-formulation (**Figure 1C**-**D**). Morphological examination by TEM further confirmed that CG self-assembled into monodisperse spherical nanoparticles (**Figure 1E**).

To elucidate the self-assembly mechanism, we performed molecular dynamics simulations. The system evolved from an initially dispersed state into an ordered aggregate within 100 ns. The average root-mean-square deviation (RMSD) stabilized at around 4.79 ± 0.37 nm after approximately 25 ns, suggesting conformational equilibrium (**Figure 1F-G**). Concurrently, the radius of gyration (Rg) decreased from ~4.3 nm to ~3.2 nm, and the solvent-accessible surface area (SASA) declined from ~425 nm^2^ to ~180 nm^2^, with both parameters plateauing after 40 ns, indicating the formation of a more compact assembly with buried hydrophobic regions (**Figure 1H-I**). Additionally, the number of hydrogen bonds within the system increased from < 10 to a stable range of 30-40 after 60 ns, underscoring the importance of hydrogen-bonding networks for structural integrity (**Figure 1J**).

Free energy landscape analysis indicated a gradual decrease in free energy as RMSD increased, with a pronounced change observed in the RMSD range of 4.6-5.2 nm, corresponding to a key structural rearrangement phase and supporting the thermodynamic stability of the assembled structure (**Figure 1K**). Physicochemical profiling confirmed the enhanced hydrophilicity of CG. The calculated logP value decreased from 5.12 for pristine CE to 2.86 for the CG conjugate, consistent with its amphiphilic nature (**Figure 1L**). Stability studies demonstrated that CG NPs retained their particle size over 14 days, indicating good storage stability (**Figure 1M**). Moreover, the nanoparticles maintained excellent colloidal stability in physiologically relevant media, including PBS (pH 7.4) supplemented with 10% fetal bovine serum or 10% rat plasma/heparin at 37 ℃, supporting their potential for *in vivo* application.

### Safety profile and toxicity attenuation of CG NPs

Given that the clinical application of CE is primarily limited by its notable hepatorenal toxicity, we systematically evaluated the safety differences between CE and its Glu-conjugated nanoform CG NPs from *in vitro* to *in vivo* settings. Initial assessment using MTT assays revealed that CG NPs exhibited significantly reduced cytotoxicity against normal hepatocyte (AML-12, THLE-2) and renal (HEK293, HK-2) cell lines compared to free CE at equivalent concentrations (**Figure S11**). This improved biocompatibility was consistent in RAW 264.7 macrophages, where CE (1.25 µM) inhibited proliferation by 12.21%, while CG NPs at the same concentration had no significant impact on cell viability (**Figure S12A**). An LDH release assay further corroborated these findings, confirming the absence of notable membrane damage for CG NPs at concentrations up to 2.5 µM (**Figure S12B**). These findings collectively indicate that Glu conjugation substantially reduces the inherent cytotoxicity of CE.

**Figure 2:**
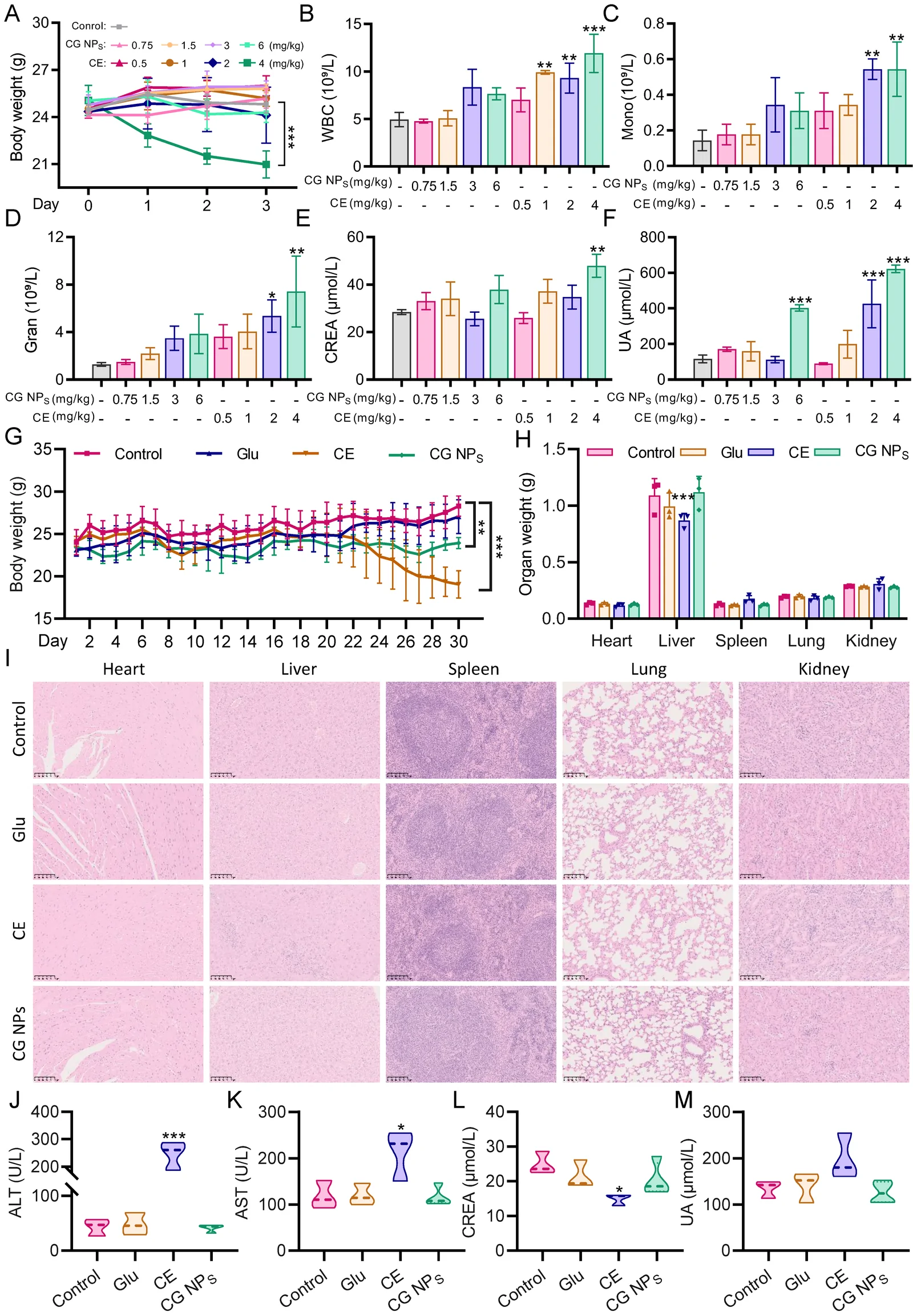
** Safety evaluation of CG NPs**. (**A**) Body weight changes in mice during the 3-day dosing period. (**B**-**D**) Hematological analysis of peripheral immune cells, measured 24 h post-final administration. (**E-F**) Serum biochemical assessment of renal function, showing levels of CREA (**E**) and UA (**F**) across different treatment cohorts. (**G**) Weight changes of mice treated with different drugs for 1 month. (**H**) Organ weights of mice in each group following the conclusion of the administration period. (**I**) H&E staining of major organs, scale bar = 100 μm. (**J**-**M**) Blood biochemical indexes of mice after treatment. All data are expressed as means ± SD of at least three independent experiments. ***P < 0.001, **P < 0.01, and *P < 0.05 vs Control group.

Subsequently, a three-day repeated-dose toxicity study in healthy mice revealed that all CG NPs-treated groups maintained stable body weight. In sharp contrast, CE at 4 mg/kg caused a significant, time-dependent decrease in body weight, suggesting acute systemic toxicity (**Figure 2A**). Furthermore, CE provoked a pronounced immune response, elevating white blood cell (WBC) counts at doses as low as 1 mg/kg and causing a comprehensive increase in WBC, monocyte (Mono), and granulocyte (Gran) counts at higher doses (2-4 mg/kg) (**Figure 2B-D**). Serum biochemistry revealed no acute hepatotoxicity (AST, ALT within normal limits) at the tested doses (**Figure S13**). However, CE displayed clear nephrotoxic potential, inducing dose-dependent elevations in UA and CREA levels at 2 and 4 mg/kg. Remarkably, CG NPs exhibited no renal toxicity at doses up to 3 mg/kg, with only a minor fluctuation in UA observed at the highest tested dose (6 mg/kg) (**Figure 2E-F**). These results established that Glu conjugation fundamentally improves the acute safety margin of CE. A one-month subchronic toxicity study was conducted to evaluate long-term risk, revealing a severe weight loss accompanied by a significant reduction in liver mass in the CE group (**Figure 2G-H**). Histopathological analysis further revealed apparent hepatocyte necrosis and immune cell infiltration in the livers of the CE-treated group (**Figure 2I**). Serum biochemical markers confirmed severe liver injury in the CE group, characterized by significantly elevated AST and ALT levels (**Figure 2J-K**). Additionally, the CE group showed altered renal parameters (decreased CREA, relative increase in UA), while all other groups remained normal (**Figure 2L-M**). Conversely, mice treated with CG NPs only exhibited slowed body weight gain (average 23.9 g). Crucially, the histology of major organs and all hepatic/renal biochemical parameters in the CG NPs group were comparable to those of the control group, with no signs of pathological damage (**Figure 2G-M**).

In summary, systematic evaluation from cells to animals consistently demonstrated that Glu conjugation markedly attenuated the cytotoxicity of CE, with CG NPs exhibiting a favorable safety profile in mice, providing a compelling experimental basis for its further development.

### Targeted delivery of CG NPs to M1 macrophages

**Figure 3:**
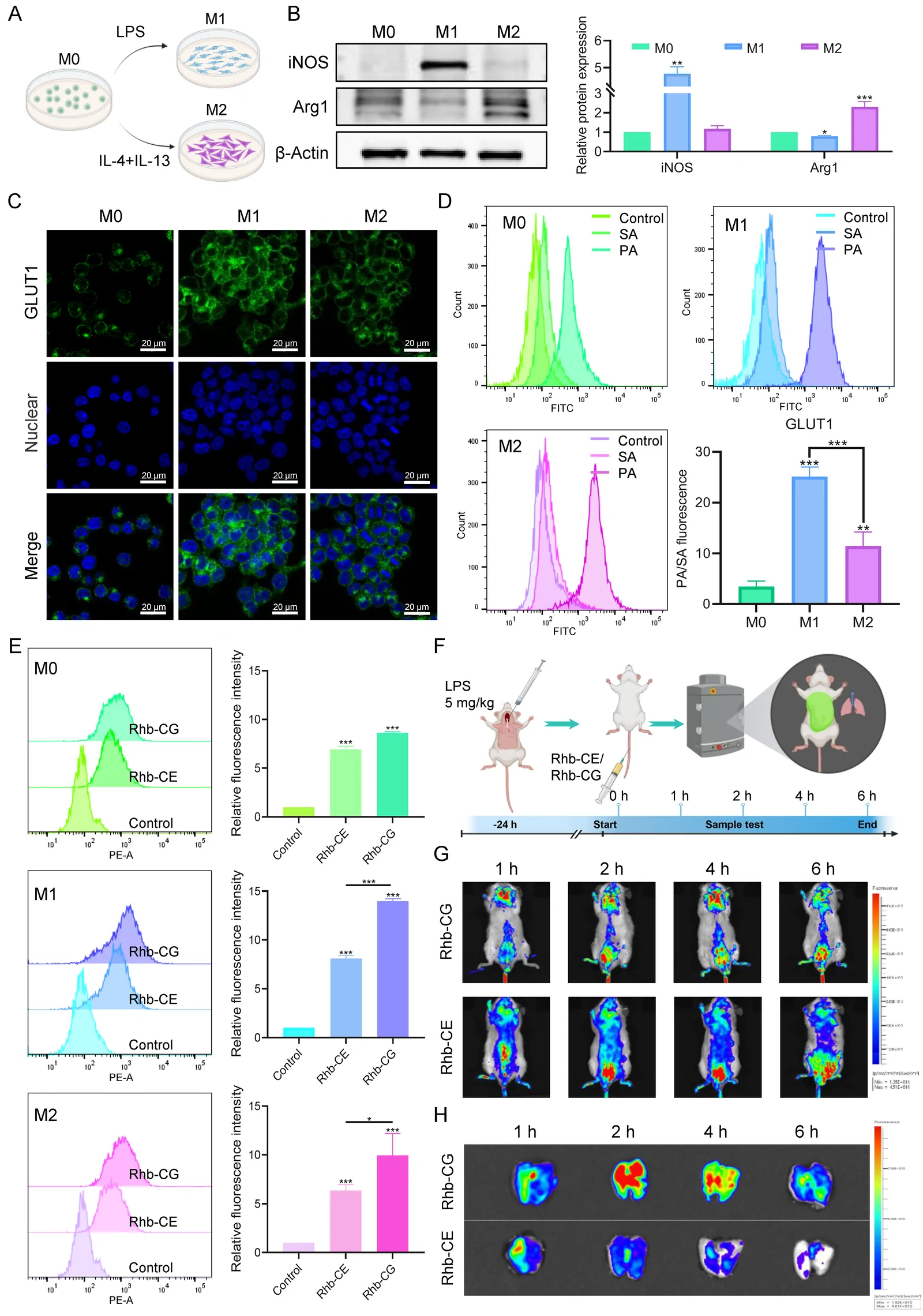
** Targeting evaluation of CG NPs *in vivo* and *in vitro.*** (**A**) Schematic representation of the macrophage polarization model. (**B**) Protein levels of iNOS and Arg1 in macrophages, analyzed by Western blot. (**C**) Representative confocal laser scanning microscopy images showing GLUT1 expression in macrophages with different polarization states (scale bar = 20 μm). (**D**) Quantitative flow cytometric analysis of GLUT1 expression in macrophage subtypes (PA represents primary antibody, SA represents secondary antibody). (**E**) Quantitative analyses of Rhb-CE and Rhb-CG uptake by macrophage subtypes via flow cytometry. (**F**) Schematic of the ALI mouse model establishment and treatment regimen. (**G**-**H**) *In vivo* biodistribution and lung retention of Rhb-CE and Rhb-CG assessed using an IVIS imaging system. Data are expressed as means ± SD of at least three independent experiments. ***P < 0.001, **P < 0.01, and *P < 0.05 vs control group.

Given their pro-inflammatory activation and metabolic reprogramming, M1 macrophages exhibit a heightened demand for Glu to fuel aerobic glycolysis, predominantly facilitated by upregulated GLUT1 expression. To explore whether CG NPs could exploit this metabolic distinction for targeted delivery, we first validated the distinct GLUT1 expression profile across macrophage polarization states using an *in vitro* model. Western blot analysis confirmed successful polarization, with LPS specifically inducing the M1 marker iNOS, and IL-4/IL-13 upregulating the M2 marker Arg1 (**Figure 3A-B**). Confocal microscopy and subsequent flow cytometric quantification revealed that GLUT1 expression was substantially higher in M1 macrophages, showing a 9.3-fold and 3.1-fold increase over M0 and M2 cells, respectively (**Figure 3C-D**). Notably, GLUT1 levels in M2 cells remained elevated compared to M0, likely reflecting their biosynthetic needs during tissue repair. These results confirmed the distinct GLUT1-high phenotype of M1 macrophages, providing a rationale for our targeting strategy.

Subsequently, we compared the cellular uptake of rhodamine-labeled CG NPs (Rhb-CG) with its non-conjugated counterpart (Rhb-CE). Flow cytometry indicated that internalization of both drugs was most pronounced in M1 macrophages. Crucially, within M1 cells, the mean fluorescence intensity of Rhb-CG was nearly twice that of Rhb-CE, directly demonstrating that Glu modification significantly enhanced drug accumulation by leveraging the high GLUT1 expression in this subset (**Figure 3E**). To confirm GLUT1-dependent uptake, a Glu competition assay was performed. Pre-incubation with excess Glu competitively inhibited the uptake of Rhb-CG but not of Rhb-CE in M1 macrophages, providing direct evidence for a GLUT1-mediated endocytosis pathway (**Figure S14**).

We next evaluated the *in vivo* targeting performance of CG NPs. Preliminary pharmacokinetic analysis showed that free CE was rapidly cleared from the bloodstream with an elimination half-life (T_1/2β_) of only 0.361 h. In contrast, Glu conjugation and self-assembled nanoformulation significantly prolonged the T_1/2β_ of CG NPs to 1.814 h, indicating enhanced systemic retention (**Table S1-2**). The plasma concentration-time curve revealed that both drugs had largely passed the initial distribution peak by 4~8 h but remained detectable, with CG NPs maintaining relatively high concentrations throughout the entire detection period. Based on these observations, we selected 6 h post-first dose as the longest observation timepoint for the subsequent *in vivo* biodistribution study in the LPS-induced ALI mouse model (**Figure 3F**, **Figure S15**). Real-time *in vivo* imaging after intravenous injection showed that Rhb-CG produced a clear pulmonary signal as early as 1 h post-injection. The signal intensified over time, peaking at 2 h and remaining elevated for up to 6 h (**Figure 3G-H**). In contrast, the fluorescence signal of Rhb-CE was initially concentrated in the mid-abdominal region at 1 h post-injection, subsequently shifting caudally with prominent accumulation in the bladder, while the pulmonary signal remained weak and declined rapidly between 2 and 6 h.

Collectively, these data demonstrated that CG NPs accumulate more rapidly and persistently in inflamed lung tissue than free CE, via a GLUT1-mediated pathway, highlighting their superior inflammatory targeting and retention capabilities.

### Therapeutic efficacy of CG NPs in ALI Mice

**Figure 4:**
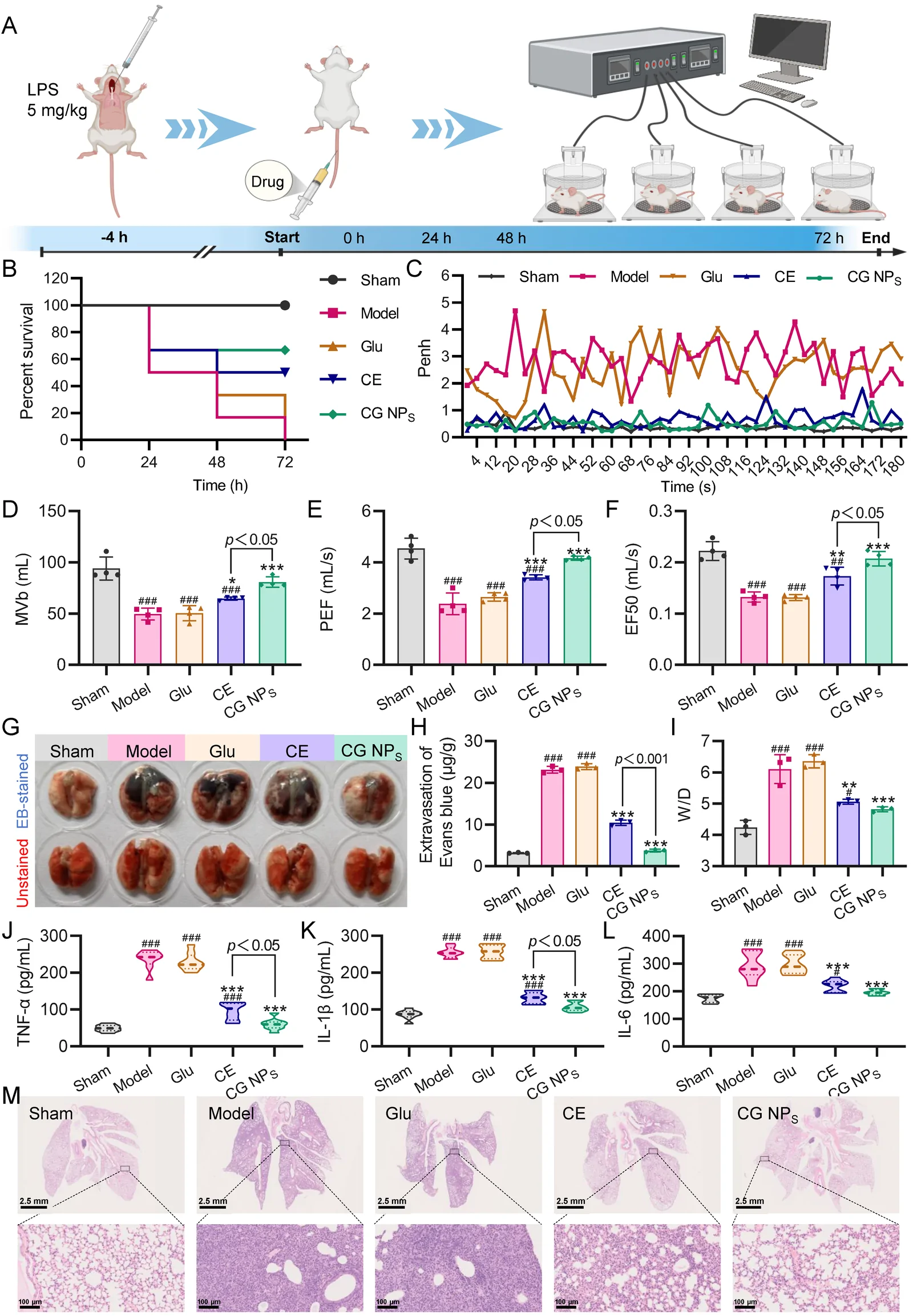
** Therapeutic effects of CG NPs on LPS-induced ALI in mice.** (**A**) Schematic illustration of ALI induction and drug administration. (**B**) Survival rates of mice across different experimental groups. (**C-F**) Pulmonary function parameters including Penh, MVb, PEF, and EF50, measured by non-invasive whole-body plethysmography at 72 h post-treatment. (**G**) Representative images of Evans blue-stained and unstained lung tissues. (**H**) Quantitative analysis of Evans blue extravasation in lung tissue. (**I**) Lung wet-to-dry weight ratio. (**J-L**) Concentrations of TNF-α, IL-1β, and IL-6 in lung tissue homogenates measured by ELISA. (**M**) Histopathological evaluation of lung tissue sections by H&E staining. All data are expressed as means ± SD from at least three independent experiments. ###*P* < 0.001, ##*P* < 0.01, and #*P* < 0.05 vs Sham group; ***P < 0.001, **P < 0.01, and *P < 0.05 vs Model group.

The marked inflammatory enrichment of CG NPs prompted a systematic evaluation of their *in vivo* therapeutic efficacy in an LPS-induced ALI mouse model (**Figure 4A**). Survival analysis revealed a 100% mortality rate within 72 h in both the model and Glu-treated groups. In contrast, the survival rates in the CE- and CG NPs-treated groups increased to 50.0% and 66.7%, respectively (**Figure 4B**). Alterations in respiratory function directly reflect the severity of lung injury. To evaluate the effect of CG NPs on ALI-associated lung function, key respiratory parameters were measured 48 h post-treatment via non-invasive whole-body plethysmography (**Figure 4C-F**). Compared with the sham group, both the model and Glu groups exhibited marked acute respiratory dysfunction, characterized by a significant elevation in the airway hyperresponsiveness index (Penh), along with pronounced reductions in MVb, PEF, and EF50, indicating severe hypoventilation and airflow obstruction in the model and Glu groups, accompanied by disruption of the lung tissue barrier and ventilation structure. These abnormalities were effectively reversed by both CE and CG NPs treatments, with reduction in Penh of 95.7% and 86.3%, respectively, as well as significant restoration of MVb, PEF, and EF50 levels. Notably, the CG NPs group demonstrated superior improvement, with respiratory parameters returning to near-baseline levels and significantly outperforming the CE group.

The extent of lung injury was further evaluated through a series of assays. The Evans blue extravasation assay and lung wet/dry weight ratio consistently indicated that both CE and CG NPs effectively attenuated LPS-induced increases in pulmonary vascular permeability and pulmonary edema, with CG NPs exhibiting a stronger protective effect than CE (**Figure 4G-I**). We then analyzed the levels of key pro-inflammatory cytokines (IL-6, TNF-α, IL-1β) in lung tissue. While CE treatment significantly suppressed their release, CG NPs induced a more potent reduction, achieving statistically greater suppression of TNF-α and IL-1β (P < 0.05). Although the superior suppression of IL-6 by CG NPs (81.8% vs. 62.0%) did not reach statistical significance, it consistently reflected a stronger inhibitory trend (**Figure 4J-L**). Histopathological examination by H&E staining provided morphological corroboration (**Figure 4M**). The model group exhibited hallmark ALI pathology, including alveolar architecture destruction, extensive inflammatory cell infiltration, and intra-alveolar hemorrhage with exudation.

Pathological injury was alleviated in the CE-treated group, with the most substantial improvement observed in the CG NPs group, where only mild alveolar septal thickening and minimal inflammatory cell infiltration were noted, closely resembling normal lung morphology.

In summary, CG NPs exerted enhanced therapeutic effect superior to free CE, through potent inhibition of inflammatory cytokine release, reduction of vascular permeability, improvement of respiratory function, and alleviation of pathological lung injury, demonstrating significant translational potential for ALI.

### *In vitro* anti-inflammatory activity of CG NPs

**Figure 5:**
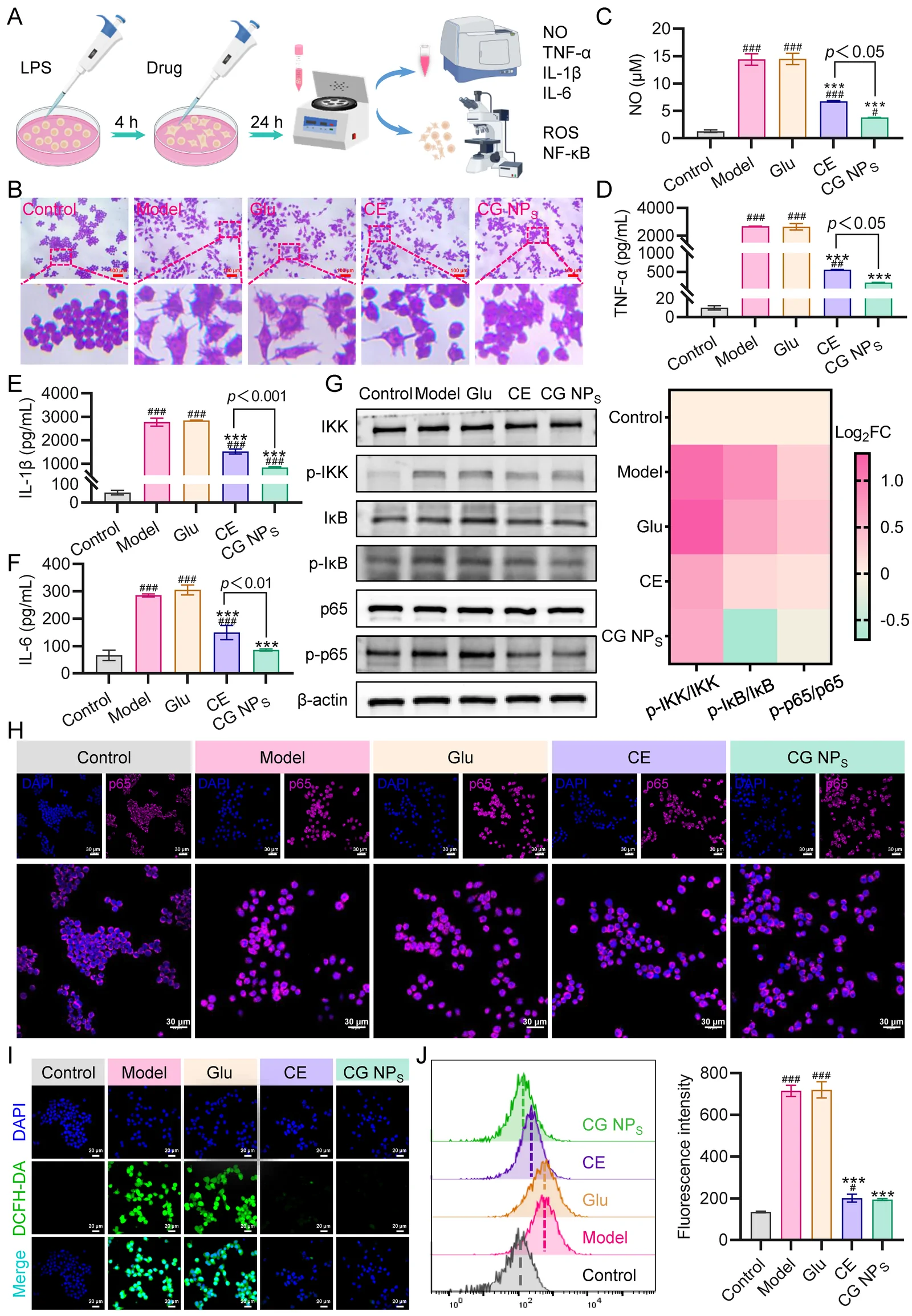
** CG NPs alleviates inflammatory responses *in vitro* through the ROS/NF-κB pathway.** (**A**) Schematic diagram of the experimental procedure. (**B**) Morphological changes of RAW 264.7 cells after co-treatment with LPS and test drugs (10X, scale bar = 100 μm). (**C**) NO levels post-treatment measured by Griess reagent. (**D-F**) The levels of TNF-α (**D**), IL-1β (**E**) and IL-6 (**F**) post-treatment detected by ELISA kit. (**G**) Protein expression and quantitative analysis related to the NF-κB pathway evaluated by Western blot analysis (log₂ fold change, normalized to control). (**H**) Nuclear translocation of P65 (purple) observed by LSCM (scale bar = 30 μm). (**I**) Intracellular ROS levels detected by LSCM (scale bar = 20 μm). (**J**) Intracellular ROS levels detected by flow cytometry. All data are expressed as means ± SD of at least three independent experiments. ###*P* < 0.001, ##*P* < 0.01, and #*P* < 0.05 vs Control group; ***P < 0.001, **P < 0.01, and *P < 0.05 vs Model group.

To elucidate the anti-inflammatory mechanism underlying the enhanced efficacy of CG NPs, we employed an LPS-stimulated RAW 264.7 macrophage model for systematic investigation (**Figure 5A**). Morphological observations showed that, unlike the model and Glu groups where cells exhibited extended pseudopodia and a differentiated state, most cells treated with CE or CG NPs maintained an oval shape with fewer pseudopods, implying effective suppression of LPS-triggered macrophage overactivation (**Figure 5B**). In terms of inflammatory mediators, both CE and CG NPs significantly attenuated LPS-induced NO production, with inhibition rates of 81.2% and 58.5%, respectively (**Figure 5C**). ELISA results further confirmed a marked reduction in the secretion of key pro-inflammatory cytokines (TNF-α, IL-1β, and IL-6), with CG NPs consistently demonstrating greater potency than CE (**Figure 5D-F**). Given the close link between pro-inflammatory cytokine release and macrophage polarization status, we further examined the regulatory effects of CG NPs on LPS-induced M1/M2 polarization in macrophages. Flow cytometry results showed that the proportion of CD86+ cells was significantly elevated in the Model group relative to the Control group, indicating successful M1 polarization by LPS. The Glu group showed a comparable proportion of CD86+ cells to the Model group, with no apparent improvement. In contrast, both the CE and CG NPs groups exhibited markedly reduced CD86+ cell percentages, suggesting that both treatments inhibited macrophage polarization toward the pro-inflammatory M1 phenotype. However, the proportion of CD206+ cells remained generally low across all groups, with minimal inter-group variation and no discernible upward trend, indicating that under the present experimental conditions, none of the treatments appreciably promoted M2 polarization (**Figure S16**).

We next focused on the NF-κB pathway, a central hub of inflammatory signaling implicated in ALI pathogenesis. Western blot analysis demonstrated that both CG NPs and CE significantly suppressed LPS-induced phosphorylation of IKKα/β, IκBα, and NF-κB p65 without altering total protein levels, with CG NPs exerting a more pronounced inhibitory effect (**Figure 5G**). This blockade of NF-κB activation was visually confirmed by confocal microscopy: while LPS stimulation caused pronounced nuclear translocation of the p65 subunit, both treatments effectively retained p65 in the cytoplasm, with CG NPs again showing greater efficacy (**Figure 5H**). Given the established crosstalk between ROS and NF-κB activation, intracellular ROS levels were further examined. Confocal imaging and flow cytometry results confirmed a significant LPS-induced ROS burst, which was effectively mitigated by both treatments. Notably, CG NPs exhibited superior ROS-scavenging capacity, reducing ROS levels by 72.8% (**Figure 5I-J**). Collectively, our findings demonstrated that CG NPs exerted potent anti-inflammatory effects compared to free CE by effectively suppressing the ROS/NF-κB signaling axis, consequently suppressing the production of NO, ROS, and pro-inflammatory cytokines.

### Modulatory effects of mitochondrial homeostasis in macrophages by CG NPs

**Figure 6:**
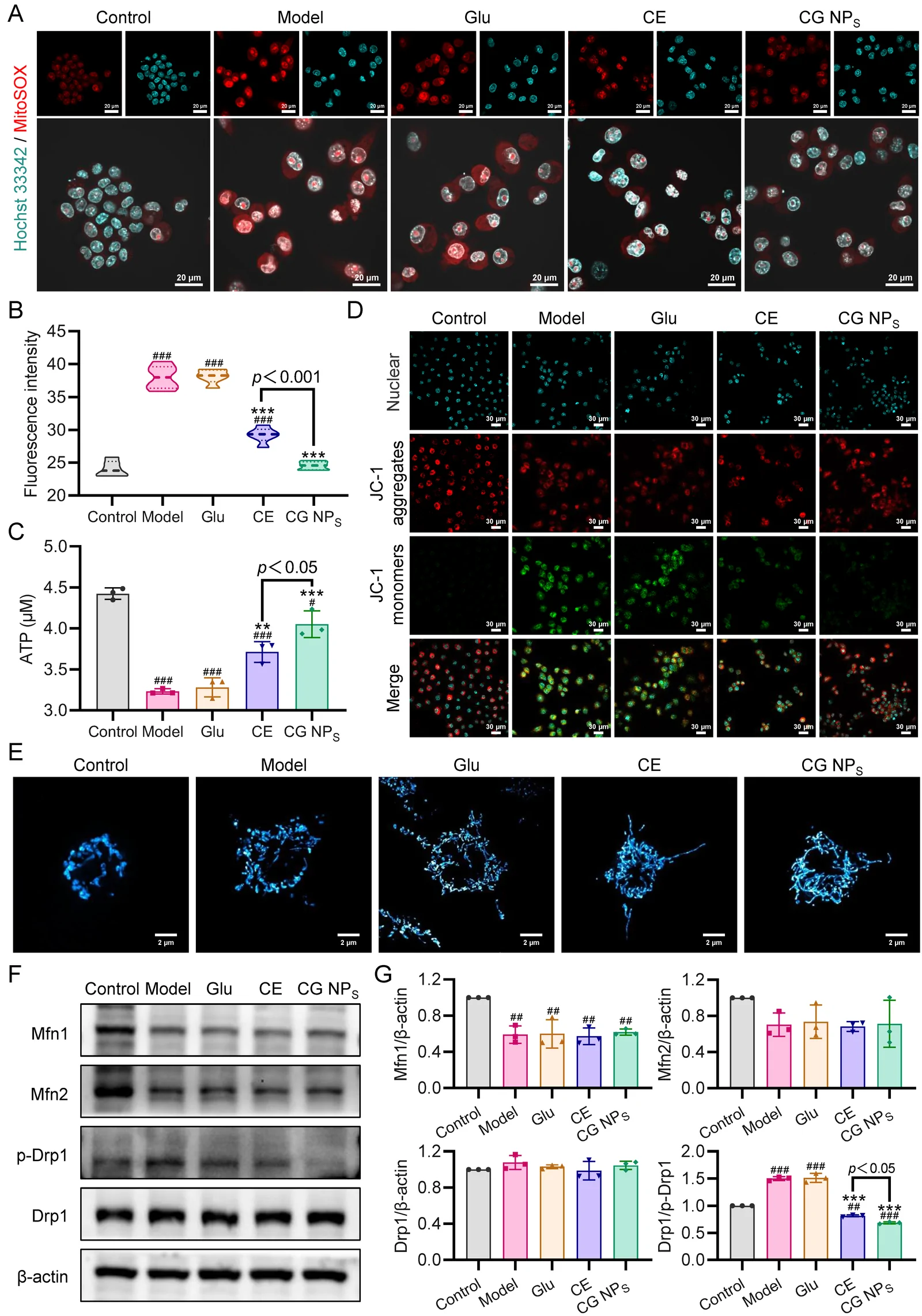
**The regulatory effect of CG NPs on macrophage mitochondria.** (**A**) MtROS levels measured by LSCM (scale bar = 20 μm). (**B**) Quantification of mtROS fluorescence intensity. (**C**) Intracellular ATP levels in RAW 264.7 cells after drug treatments. (**D**) MMP changes after drug treatments detected by JC-1 staining (scale bar = 30 μm). (**E**) The morphological changes of mitochondria observed by super-resolution microscope (scale bar = 2 μm). (**F**) The protein expression levels of Mfn1, Mfn2, Drp1 and p-Drp1 in RAW 264.7 cells detected by Western blot. (**G**) Quantification of Mfn1, Mfn2, Drp1, and p-Drp1 protein levels. All data are expressed as means ± SD of at least three independent experiments. ###*P* < 0.001, ##*P* < 0.01, and #*P* < 0.05 vs Control group; ***P < 0.001, **P < 0.01, and *P < 0.05 vs Model group.

Previous studies have established the role of CG NPs in suppressing intracellular ROS. Given that mitochondria generate ~90% of cellular ROS, we measured mitochondrial-derived ROS (mtROS) via confocal microscopy 33. LSCM showed that both CG NPs and CE significantly attenuated LPS-induced mtROS overproduction, confirming their targeted mitigation of mitochondrial oxidative stress (**Figure 6A-B**). Given the vicious cycle between mitochondrial dysfunction and inflammation, we next systematically evaluated whether CG NPs preserve cellular homeostasis through restoring mitochondrial function by assessing three key parameters: bioenergetics, membrane integrity, and network morphology. LPS stimulation severely impaired ATP production, and this deficit was more effectively reversed by CG NPs than by free CE, indicating superior restoration of mitochondrial energy metabolism (**Figure 6C**). Using JC-1 staining, we found that both treatments significantly prevented LPS-induced MMP depolarization, as shown by increased JC-1 aggregates (red) and decreased monomers (green), reflecting preserved mitochondrial health (**Figure 6D**). Super-resolution microscopy further revealed that LPS stimulation caused profound mitochondrial fragmentation, transforming the tubular network into punctate granules, whereas both treatments effectively preserved elongated mitochondrial morphology, indicating a protective effect on mitochondrial network integrity (**Figure 6E**).

Since mitochondrial shape is dynamically regulated by fission and fusion, we examined the expression of key regulatory proteins. Western blot analysis showed that while CG NPs and CE did not significantly alter the levels of the fusion proteins Mfn1 and Mfn2, both markedly inhibited the phosphorylation of the fission protein Drp1, indicating that their protective effect is primarily mediated by restraining Drp1-driven excessive fission (**Figure 6F-G**).

Collectively, this study established the maintenance of mitochondrial function and integrity as a pivotal mechanism for the anti-inflammatory activity of CG NPs, offering a novel dimension to the understanding of their pharmacology.

### Mitochondrial fission inhibition of CG NPs via Drp1 targeting

**Figure 7:**
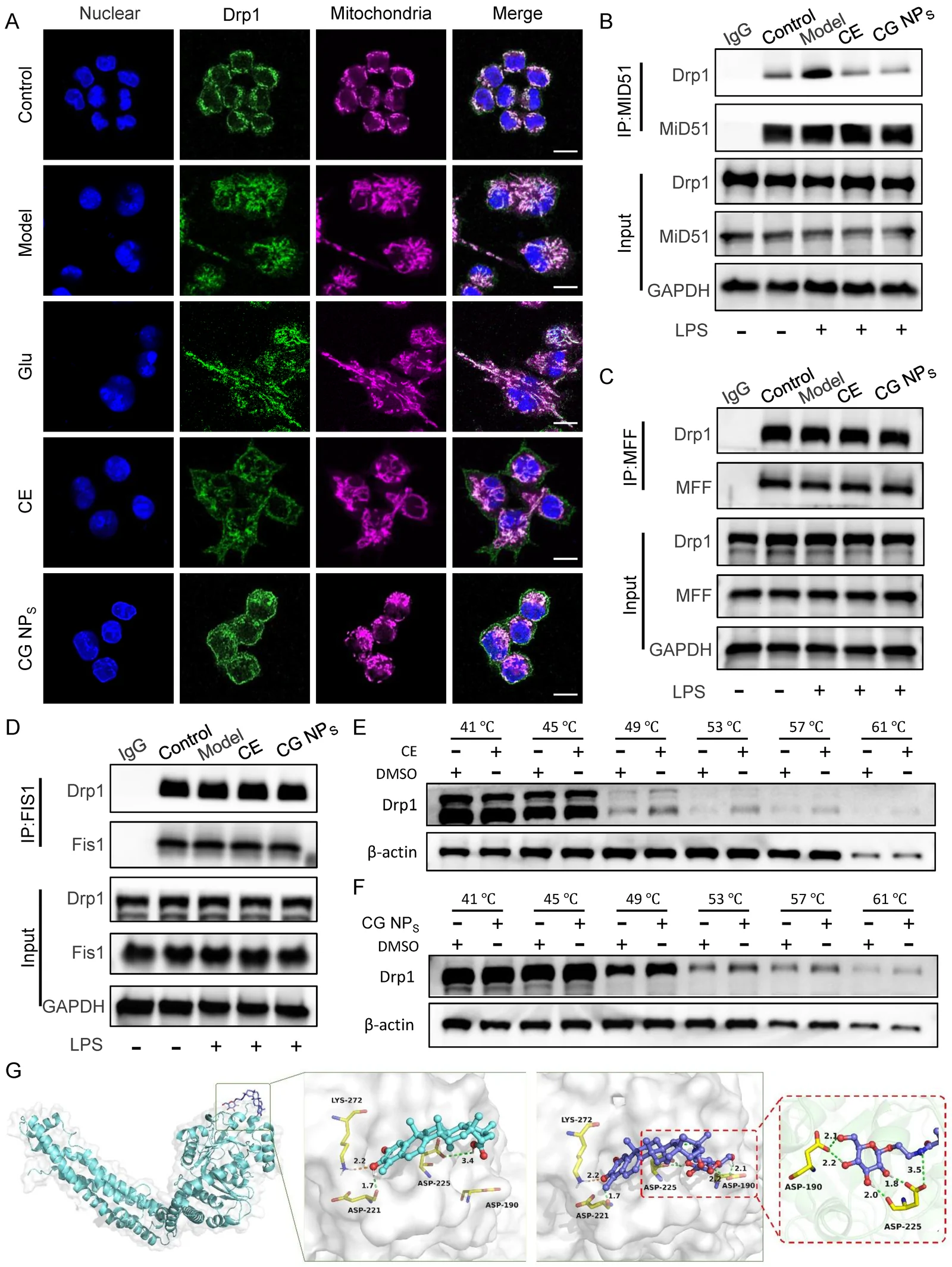
** CG NPs suppress mitochondrial excessive fission by targeting Drp1.** (**A**) Representative confocal images showing the co-localization of Drp1 (green) with mitochondria (purple) (scale bar = 10 μm). (**B**-**D**) Co-IP analysis of the interactions between Drp1 and mitochondrial fission receptors, including (**B**) MiD51, (**C**) MFF, and (**D**) Fis1, in RAW 264.7 cells. (**E**, **F**) CETSA analysis of Drp1 thermal stability in response to CE (**E**) or CG NPs (**F**) treatment. (**G**) Molecular docking models of CE and CG in complex with Drp1.

Building on the previous findings that CG NPs inhibit Drp1 activation and preserve mitochondrial network integrity, we further dissected the molecular mechanism underlying their suppression of excessive mitochondrial fission. We first assessed their impact on the mitochondrial translocation of Drp1, a requisite step for fission initiation whereby Drp1 relocates from the cytosol to the outer mitochondrial membrane. Confocal microscopy revealed that, compared to the model group, treatment with either CG NPs or CE markedly diminished the punctate accumulation of Drp1 on mitochondria while increasing its diffuse distribution in the cytosol, indicating effective blockade of Drp1 mitochondrial translocation (**Figure 7A**). Given that mitochondrial recruitment of Drp1 depends on its interaction with specific outer-membrane receptors, we evaluated the binding of Drp1 to its major receptors (MiD51, Fis1, MFF) by co-immunoprecipitation (Co-IP). LPS stimulation selectively enhanced the Drp1-MiD51 interaction without significantly affecting its binding to Fis1 or MFF (**Figure 7B-D**). Notably, both CG NPs and CE treatment substantially attenuated the formation of the Drp1-MiD51 complex, while leaving other Drp1-receptor interactions unaltered, suggesting that specifically disrupting Drp1-MiD51 binding impairs Drp1 recruitment. To investigate whether CG NPs and CE bind directly to Drp1, a cellular thermal shift assay (CETSA) was employed. Under a thermal gradient (41-61 ℃), Drp1 in the control (DMSO) group underwent progressive denaturation and precipitation, leading to a sharp decline in soluble protein levels. In contrast, pretreatment with CG NPs or CE significantly increased the amount of residual soluble Drp1, confirming direct binding and enhanced thermal stability of the protein (**Figure 7E-F**).

The binding affinity of a small molecule to a protein is largely determined by their structural compatibility and the ensuing network of interactions 34. To map these details, we performed molecular docking. The results revealed that the natural product CE was well-embedded within the GTPase domain of Drp1, forming notable interactions with residues such as ASP-221, LYS-272, and ASP-225. Upon introduction of the Glu moiety, CG adopted a distinct U-shaped conformation and deeply inserted into the protein pocket of Drp1. While maintaining the original binding interactions with ASP-221 and LYS-272, its glycosyl group appeared to form a dense hydrogen-bonding network with ASP-225 and ASP-190, which may contribute to enhanced binding affinity (**Figure 7G**). Notably, ASP-221 and ASP-190 are considered core contacts for Drp1 anchoring to mitochondrial receptors, mediating initial recruitment through critical salt bridges with MiD51. We speculate that CE likely produces steric occlusion by physically occupying ASP-190, partially blocking Drp1-MiD51 interaction. In comparison, CG may competitively occupy the core interface involving ASP-190 and ASP-221, potentially imposing steric hindrance and, through its dense hydrogen-bonding network, perturbing the local electrostatic microenvironment. These *in silico* findings suggest that CG could disrupt the specific recognition between Drp1 and MiD51 more efficiently than CE at both steric and electrostatic levels.

This study confirmed that CE could directly bind to Drp1 and competitively inhibit the Drp1-MiD51 interaction, while Glu conjugation potentially enhances the binding affinity and spatial complementarity of CG toward Drp1 through a denser hydrogen-bond network, thereby more effectively blocking Drp1 mitochondrial recruitment and fission complex assembly. Consequently, CG NPs exhibited stronger effects in maintaining mitochondrial homeostasis, mitigating oxidative stress, and ultimately delivering superior anti-inflammatory efficacy.

## Conclusion

This study developed a glucose-modified CE prodrug that self-assembled into carrier-free nanoparticles (CG NPs), effectively overcoming the clinical translational barriers of CE. The CG NPs formed a homogeneous and stable nanostructure driven by synergistic hydrophobic interactions and hydrogen-bonding networks. Glucose conjugation significantly mitigated the inherent hepatorenal toxicity of CE, thereby widening its therapeutic window. Exploiting the high GLUT1 expression characteristic of metabolically reprogrammed pro-inflammatory M1 macrophages, CG NPs achieved active targeting and efficient internalization by this cell subset.

In an LPS-induced ALI model, CG NPs demonstrated rapid and persistent accumulation at inflammatory pulmonary sites. *In vivo* evaluation revealed that CG NPs significantly improved the survival rate, ameliorated respiratory function, reduced pulmonary edema and vascular permeability, and markedly attenuated pathological lung injury in ALI mice, showcasing superior overall therapeutic efficacy compared to free CE. Mechanistically, CG NPs suppressed excessive macrophage activation by inhibiting the ROS/NF-κB inflammatory signaling axis. Further investigation identified Drp1, a key mediator of mitochondrial fission, as the primary target. CG NPs directly bind to Drp1, competitively disrupting the interaction with its receptor MiD51, thereby suppressing excessive mitochondrial fission and maintaining mitochondrial network integrity, energy metabolism, and redox homeostasis. Mitochondrial biogenesis and mitophagy are also known to play critical roles in maintaining mitochondrial homeostasis. Although not directly assessed in this study, the restoration of ATP levels and reduction of mtROS by CG NPs may create a favorable metabolic environment for PGC-1α-driven biogenesis, and the inhibition of excessive Drp1-mediated fission could help preserve physiological mitophagy flux. Molecular docking suggested that glucose conjugation may enhance the binding affinity and spatial compatibility of the prodrug with Drp1, which could contribute to the greater potency of CG NPs compared with free CE in inhibiting mitochondrial fragmentation and the subsequent inflammatory cascade. We acknowledge that quantitative binding studies (e.g., surface plasmon resonance or isothermal titration calorimetry) would be needed to validate these *in silico* findings. These possibilities also warrant further investigation.

In conclusion, this work established a novel nanoprodrug paradigm that concurrently enables precise inflammatory-cell targeting and mitochondrial protection. By leveraging the metabolic signature (GLUT1 overexpression) of M1 macrophages for targeted delivery and directly intervening in a key mitochondrial dynamics node (Drp1) to reestablish cellular homeostasis, this strategy offers a promising therapeutic avenue for ALI and other inflammatory diseases.

## Supplementary Material

Supplementary figures and tables.

## Abbreviations

ALI: acute lung injury
ALT: alanine aminotransferase
AST: aspartate aminotransferase
CE: celastrol
CG: glucose-conjugated CE
CREA: creatinine
DLS: dynamic light scattering
Drp1: Dynamin-related protein 1
EF50: expiratory flow at 50 % of tidal volume
GLUT1: glucose transporter 1
Gran: neutrophil count
LPS: lipopolysaccharide
Mono: monocyte count
mtROS: mitochondrial reactive oxygen species
LSCM: laser scanning confocal microscope
MVb: minute ventilation
PDI: polydispersity index
PEF: peak expiratory flow
Penh: Enhanced Pause
ROS: reactive oxygen species
TEM: transmission electron microscopy
UA: uric acid
WBC: white blood cell count
