# Supplementary material for: Celastrol-Based Hybrid Prodrug Ameliorates ALI by Spatiotemporally Consecutive Dual-Targeting GLUT1/Drp1 to Reestablish Mitochondrial Homeostasis
Source: Int J Biol Sci. 2026 Aug 21;22(13):7427–46. doi: 10.7150/ijbs.133756 (PMC13540998; doi:10.7150/ijbs.133756)
Supplement: Supplementary file 1 [file ijbsv22p7427s1.pdf]

Supporting information

1. Compounds synthesis

Synthesis of compound 2

β-D-Glucose pentaacetate (compound **1**, 5.00 g, 12.82 mmol) was dissolved in dichloromethane (DCM, 70 mL) in a 100 mL round-bottom flask. After adding Fmoc-Gly-ol (1.83 g, 6.40 mmol), the mixture was cooled to 0 °C, followed by dropwise addition of boron trifluoride etherate (2.28 g, 19.23 mmol). The reaction was stirred overnight at room temperature, with progress monitored by TLC (petroleum ether/ethyl acetate = 1:1) and LC-MS. After completion, the mixture was diluted with a large volume of DCM and washed three times with saturated NaCl solution. The combined organic layers were dried over anhydrous sodium sulfate, filtered, and concentrated under reduced pressure. Purification by column chromatography (petroleum ether/ethyl acetate = 2:1) afforded compound **2** as a light-yellow solid (6.32 g, 81%). ^{1}H NMR (600 MHz, $CDCl_3$) δ 7.77 (t, J = 7.6 Hz, 2H), 7.60 (t, J = 7.4 Hz, 2H), 7.41 (dt, J = 11.5, 7.4 Hz, 2H), 7.32 (tdd, J = 7.4, 4.8, 1.2 Hz, 2H), 5.26 – 5.19 (m, 2H), 5.04 (t, J = 9.7 Hz, 1H), 4.97 (dd, J = 9.7, 8.0 Hz, 1H), 4.57 (dd, J = 10.8, 6.4 Hz, 1H), 4.43 (dd, J = 10.7, 6.3 Hz, 1H), 4.35 (d, J = 8.0 Hz, 1H), 4.20 (t, J = 6.4 Hz, 1H), 4.16 – 4.07 (m, 2H), 3.74 (dq, J = 56.1, 5.4 Hz, 2H), 3.36 (q, J = 5.5 Hz, 2H), 2.08 – 1.97 (m, 12H). ^{13}C NMR (151 MHz, $CDCl_3$) δ 170.55, 170.26, 169.48, 169.39, 156.41, 143.94, 143.83, 141.37, 141.32, 127.76, 127.70, 127.11, 127.06, 125.01, 124.86, 120.02, 120.00, 101.24, 72.59, 71.81, 71.28, 70.00, 68.33, 66.16, 61.88, 47.37, 41.07, 20.62, 20.60. HRMS (ESI) *m/z* caled for $C_{31}H_{35}NO_{12}$ $[M+Na]^+$: 636.6058.

Synthesis of compound 3

A solution of compound **2** (4.00 g, 6.53 mmol) in MeOH (20 mL) was treated with KOH (0.54 g, 9.80 mmol) and stirred at room temperature for 4 h, with progress monitored by TLC (petroleum ether/ethyl acetate = 1:1) and LC-MS. The methanol was then removed under reduced pressure, and the residue was dissolved in a large volume of water. The aqueous phase was adjusted to pH = 7 with 0.2 M HCl and extracted three times with ethyl acetate. The combined aqueous layers were concentrated under reduced pressure, and the crude product was taken up in anhydrous EtOH, filtered, and concentrated to give compound **3** as a yellow oil (0.94 g, 65%). ^{1}H NMR (700 MHz, Methanol-d_4) δ 4.38 (d, J = 7.8 Hz, 1H), 4.10 – 4.06 (m, 1H), 3.91 (ddd, J = 17.0, 9.6, 4.1 Hz, 2H), 3.69 (dd, J = 11.9, 5.8 Hz, 2H), 3.44 – 3.41 (m, 1H), 3.32 – 3.31 (m, 1H), 3.27 (dd, J = 9.3, 7.8 Hz, 1H), 3.21 (dd, J = 6.1, 3.8 Hz, 2H). ^{13}C NMR (151 MHz, MeOD) δ 102.78, 76.62, 76.37, 73.55, 70.09, 65.59, 61.12, 48.56. HRMS (ESI) *m/z* caled for $C_8H_{17}NO_6$ $[M+H]^+$: 224.1136.

Synthesis of CG

A solution of CE (0.20 g, 0.44 mmol) in DMF (5 mL) was treated sequentially with HATU (0.20 g, 0.53 mmol) and

DIPEA (0.11 g, 0.88 mmol). After stirring for 30 min, compound **3** (0.20 g, 0.88 mmol) was added, and stirring continued at room temperature for 4 h with reaction monitored by TLC (DCM/MeOH = 10:1) and LC–MS. Upon completion, the mixture was extracted with DCM and washed with brine. The organic phase was dried (Na_2SO_4), concentrated, and purified by column chromatography (DCM/MeOH = 25:1) to yield CE-Glu as an orange-yellow solid (0.21 g, 70%). ^{1}H NMR (600 MHz, Methanol-*d*$_4$) δ 7.72 (dd, *J* = 5.7, 3.3 Hz, 1H), 7.61 (d, *J* = 2.5 Hz, 1H), 6.49 – 6.46 (m, 1H), 4.29 (s, 2H), 4.23 (d, *J* = 7.9 Hz, 1H), 3.86 – 3.79 (m, 2H), 3.59 (td, *J* = 9.5, 7.2, 4.2 Hz, 2H), 3.37 (t, *J* = 4.5 Hz, 1H), 3.24 (d, *J* = 5.4 Hz, 2H), 3.19 – 3.17 (m, 1H), 2.48 (d, *J* = 15.8 Hz, 1H), 2.26 (d, *J* = 14.4 Hz, 1H), 2.21 (s, 3H), 2.12 (d, *J* = 9.9 Hz, 2H), 1.94 – 1.90 (m, 2H), 1.86 – 1.83 (m, 1H), 1.74 – 1.70 (m, 5H), 1.65 (d, *J* = 7.9 Hz, 1H), 1.61 (s, 1H), 1.47 (d, *J* = 1.9 Hz, 3H), 1.31 (s, 3H), 1.14 (s, 3H), 0.98 (s, 3H), 0.89 (d, *J* = 7.2 Hz, 1H), 0.65 (d, *J* = 4.9 Hz, 3H). ^{13}C NMR (151 MHz, MeOD) δ 179.63, 178.72, 171.01, 165.16, 146.53, 135.11, 127.21, 119.15, 118.76, 118.34, 103.08, 76.69, 73.75, 70.39, 67.79, 61.36, 44.93, 44.51, 42.88, 39.99, 39.97, 39.47, 39.31, 37.49, 36.21, 34.71, 33.60, 32.74, 30.72, 30.65, 30.36, 29.42, 29.04, 28.38, 20.76, 17.93, 8.91. HRMS (ESI) *m/z* caled for $C_{37}H_{53}NO_9$ $[M+H]^+$: 656.3811.

Synthesis of Rhodamine B-1

A mixture of Rhodamine B (1.00 g, 2.09 mmol) and 1,2-ethanediamine (0.15 g, 3.13 mmol) in anhydrous EtOH (20 mL) was heated under reflux for 12 h. The solvent was then evaporated in vacuo. The residue was extracted with DCM, and the organic layer was dried over anhydrous sodium sulfate, filtered, and concentrated. The crude product was purified by column chromatography (DCM/MeOH = 20:1) to afford Rhodamine B-1 as a pink solid (0.62 g, 61% yield). ^{1}H NMR (600 MHz, $CDCl_3$) δ 7.87 (dd, *J* = 5.9, 2.9 Hz, 1H), 7.44 – 7.39 (m, 2H), 7.07 (dd, *J* = 5.9, 2.7 Hz, 1H), 6.46 – 6.21 (m, 6H), 3.41 – 3.16 (m, 10H), 3.02 (s, 2H), 2.50 (t, *J* = 6.3 Hz, 2H), 1.15 (t, *J* = 7.1 Hz, 12H). ^{13}C NMR (151 MHz, $CDCl_3$) δ 168.87, 153.53, 153.26, 148.82, 132.50, 130.94, 128.59, 128.06, 123.81, 122.76, 108.19, 105.29, 97.72, 65.17, 44.32, 42.96, 40.72, 12.59. HRMS (ESI) *m/z* caled for $C_{30}H_{36}N_4O_2$ $[M+H]^+$: 485.2864.

Synthesis of CE-1

A mixture of CE (1.00 g, 2.22 mmol), octanedioic acid (1.54 g, 8.88 mmol), DIC (0.34 g, 2.67 mmol), TEA (0.22 g, 2.22 mmol), and DMAP (0.14 g, 1.12 mmol) in DMF (10 mL) was stirred at room temperature for 8 h, with reaction monitored by TLC until completion. The mixture was then diluted with DCM, and the organic layer was separated, dried over anhydrous sodium sulfate, filtered, and concentrated under reduced pressure. The crude product was purified by column chromatography (DCM/MeOH = 15:1) to afford the target compound CE-1 as a yellow solid (0.82 g, 68% yield). ^{1}H NMR (600 MHz, $CDCl_3$) δ 7.08 – 7.06 (m, 1H), 6.53 (d, *J* = 1.4 Hz, 1H), 6.32 (d, *J* = 7.2 Hz, 1H), 2.60 (td, *J* = 7.5, 2.3 Hz, 2H), 2.46 – 2.41 (m, 2H), 2.34 (td, *J* = 7.5, 4.4 Hz, 3H), 2.13 (s, 5H), 1.84 (d, *J* = 3.9 Hz, 2H), 1.75 (t, *J* = 7.5 Hz, 2H),

1.64 (t, J = 7.4 Hz, 5H), 1.58 (d, J = 8.0 Hz, 2H), 1.46 (s, 3H), 1.39 (d, J = 6.8 Hz, 4H), 1.26 (d, J = 3.3 Hz, 5H), 1.22 (s, 3H), 1.19 (d, J = 6.5 Hz, 3H), 1.09 (s, 3H), 0.68 (s, 3H). ^{13}C NMR (151 MHz, $CDCl_3$) δ 184.46, 179.45, 177.67, 172.47, 171.42, 163.39, 142.92, 135.83, 133.86, 126.29, 122.83, 117.85, 45.34, 44.20, 42.83, 40.23, 39.27, 38.28, 36.33, 34.61, 33.98, 33.73, 33.53, 32.67, 31.55, 30.81, 30.54, 29.48, 29.46, 28.79, 28.73, 28.63, 28.59, 28.55, 25.03, 24.71, 24.44, 22.56, 21.96, 20.97, 18.86, 11.37. HRMS (ESI) *m/z* caled for $C_{37}H_{50}O_7$ $[M+ Na]^+$: 629.3452.

Synthesis of CE-2

A solution of CE-1 (0.50 g, 0.83 mmol), HATU (0.375 g, 1.00 mmol), and DIPEA (0.225 g, 1.65 mmol) in DCM (10 mL) was stirred at room temperature for 30 min. Rhodamine B-1 (0.40 g, 0.83 mmol) was then added, and the reaction was monitored by TLC until complete. Subsequently, the mixture was washed with brine, and the organic layer was dried over anhydrous sodium sulfate, filtered, and concentrated under reduced pressure. Purification of the crude product by column chromatography (DCM/MeOH = 20:1) afforded the target compound CE-2 as a light red solid (0.45 g, 63% yield). ^{1}H NMR (600 MHz, $CDCl_3$) δ 7.92 – 7.89 (m, 1H), 7.45 (dd, J = 5.7, 3.1 Hz, 2H), 7.09 – 7.02 (m, 2H), 6.81 (d, J = 4.7 Hz, 1H), 6.53 (s, 1H), 6.42 (d, J = 8.8 Hz, 2H), 6.37 (d, J = 2.6 Hz, 2H), 6.30 – 6.25 (m, 3H), 3.33 (qd, J = 7.2, 2.2 Hz, 8H), 3.29 (dd, J = 6.3, 3.5 Hz, 2H), 3.05 (q, J = 4.7 Hz, 2H), 2.59 (t, J = 7.5 Hz, 2H), 2.43 (d, J = 15.8 Hz, 1H), 2.12 (s, 4H), 2.10 – 2.02 (m, 3H), 1.84 (dd, J = 12.7, 7.9 Hz, 2H), 1.76 – 1.72 (m, 2H), 1.67 (d, J = 7.7 Hz, 2H), 1.58 (q, J = 9.1, 8.3 Hz, 4H), 1.53 (s, 1H), 1.44 (s, 3H), 1.43 – 1.39 (m, 2H), 1.36 – 1.31 (m, 3H), 1.25 (d, J = 3.1 Hz, 4H), 1.17 (q, J = 7.2 Hz, 17H), 1.08 (s, 3H), 0.97 – 0.92 (m, 1H), 0.67 (s, 3H). ^{13}C NMR (151 MHz, $CDCl_3$) δ 180.53, 175.68, 171.20, 170.21, 169.38, 167.97, 161.21, 151.87, 151.32, 146.97, 140.96, 133.50, 131.68, 130.83, 128.48, 126.48, 126.23, 124.36, 121.94, 120.91, 120.89, 115.79, 106.31, 102.81, 95.84, 63.73, 43.34, 42.40, 42.21, 40.81, 38.54, 38.32, 38.18, 37.25, 36.29, 34.61, 34.34, 32.69, 31.88, 31.48, 30.77, 29.61, 28.80, 28.57, 27.74, 27.61, 27.49, 26.95, 26.91, 26.68, 23.56, 22.89, 20.07, 17.04, 10.65, 9.41. HRMS (ESI) *m/z* caled for $C_{67}H_{84}N_4O_8$ $[M+ H]^+$: 1073.6353.

Synthesis of Rhb-CG

A mixture of CE-2 (0.10 g, 0.09 mmol) and Glu (0.04 g, 0.19 mmol) in DMF was treated with HATU (0.04 g, 0.12 mmol) and DIPEA (0.03 g, 0.19 mmol). After stirring at 25 °C for 2 h (monitored by TLC), the reaction was worked up by extraction with DCM. The organic phase was dried over anhydrous sodium sulfate and concentrated under reduced pressure. Purification by column chromatography (DCM/MeOH = 15:1) yielded Rhb-CE-Glu as a red solid (0.12 g, 41%). ^{1}H NMR (600 MHz, $CDCl_3$) δ 7.84 – 7.81 (m, 1H), 7.39 – 7.36 (m, 2H), 7.12 – 6.99 (m, 2H), 6.78 (t, J = 4.6 Hz, 2H), 6.45 (s, 1H), 6.36 – 6.33 (m, 2H), 6.31 (d, J = 2.5 Hz, 3H), 6.20 (dt, J = 9.1, 2.4 Hz, 2H), 4.19 (d, J = 7.6 Hz, 1H), 3.74 (t, J = 16.8 Hz, 2H), 3.67 – 3.59 (m, 1H), 3.59 – 3.53 (m, 1H), 3.49 (d, J = 8.9 Hz, 1H),

3.41 (d, J = 13.2 Hz, 2H), 3.27 – 3.25 (m, 6H), 3.21 (t, J = 5.4 Hz, 2H), 3.09 (d, J = 7.4 Hz, 1H), 2.96 (q, J = 5.2 Hz, 1H), 2.89 (s, 1H), 2.81 (s, 1H), 2.50 (d, J = 7.5 Hz, 2H), 2.30 – 2.23 (m, 2H), 2.16 – 2.12 (m, 1H), 2.08 (s, 3H), 2.01 (t, J = 7.7 Hz, 3H), 1.84 – 1.76 (m, 2H), 1.68 – 1.59 (m, 4H), 1.53 – 1.47 (m, 4H), 1.44 – 1.34 (m, 10H), 1.26 (s, 4H), 1.20 (d, J = 18.2 Hz, 12H), 1.09 (t, J = 7.0 Hz, 12H), 1.06 (s, 2H), 1.01 (s, 2H), 0.83 – 0.76 (m, 2H), 0.56 (s, 2H). ^{13}C NMR (151 MHz, $CDCl_3$) δ 177.83, 176.69, 172.44, 170.73, 168.85, 161.60, 152.78, 152.27, 147.93, 141.83, 131.86, 129.33, 127.38, 127.23, 124.89, 122.91, 121.81, 121.29, 107.24, 103.61, 101.98, 96.80, 74.85, 72.61, 69.74, 64.76, 53.98, 44.48, 43.64, 43.34, 42.21, 42.07, 39.14, 38.54, 37.27, 35.50, 35.44, 33.99, 33.85, 32.91, 32.75, 30.90, 30.68, 30.44, 30.41, 29.57, 29.17, 29.04, 28.71, 28.68, 28.64, 28.34, 27.81, 27.67, 24.41, 23.75, 21.67, 20.71, 17.61, 17.41, 13.10, 11.59, 11.38, 10.38. HRMS (ESI) *m/z* caled for $C_{75}H_{99}N_5O_{13}$ $[M+H]^+$: 1278.7288.

2. Synthetic Schemes

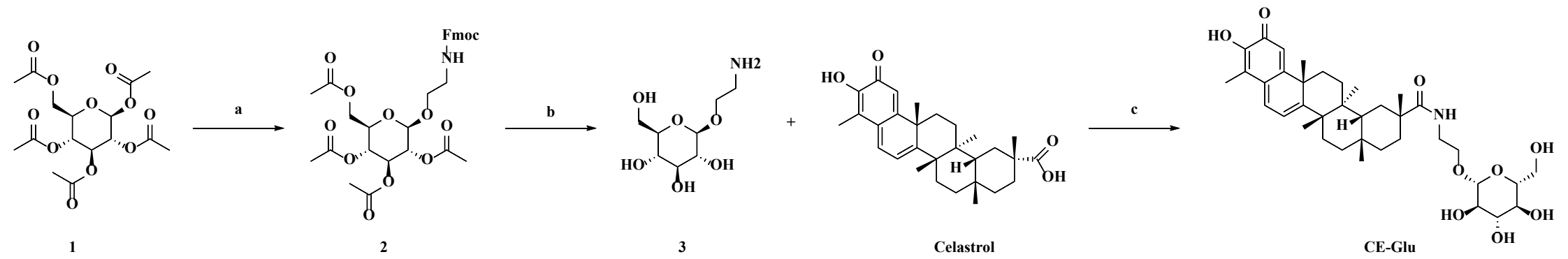

Scheme S1 Synthesis of CE-Glu. (a) Fmoc-Gly-ol, Boron trifluoride etherate, DCM, 0 °C to rt, 12 h. (b) KOH, MeOH, 1 h; (c) HATU, DIPEA, DMF, rt, 4 h.

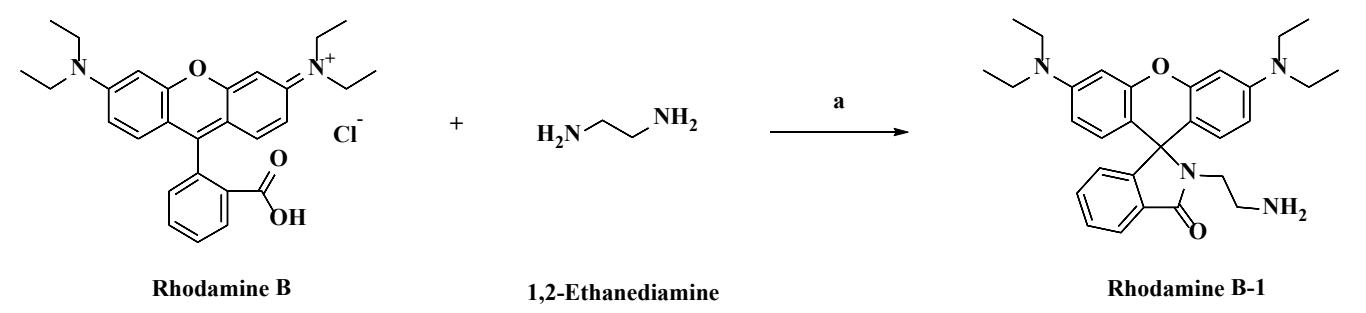

Scheme S2 Synthesis of Rhodamine B-1. (a) EtOH, 60 °C, 12 h.

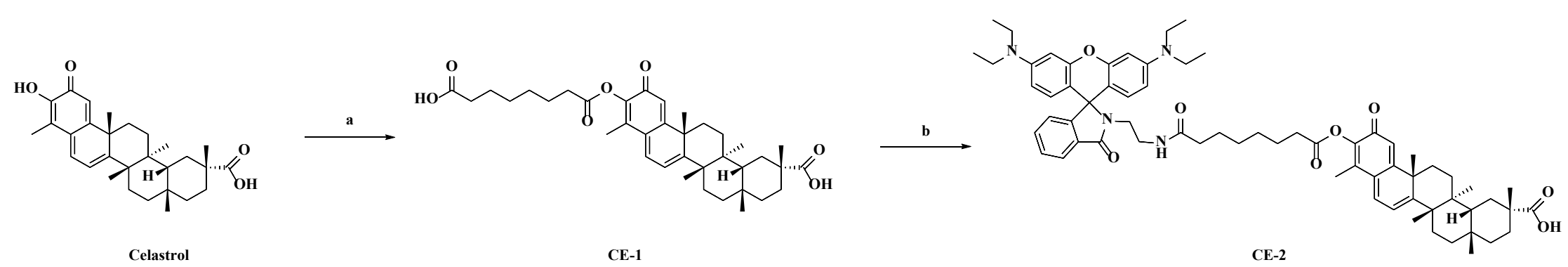

Scheme S3 Synthesis of CE-2. (a) Octanedioic acid, DIC, TEA, DMAP, DMF, rt, 6 h. (b) Rhodamine B-1, HATU, DIPEA, DCM, rt, 2 h.

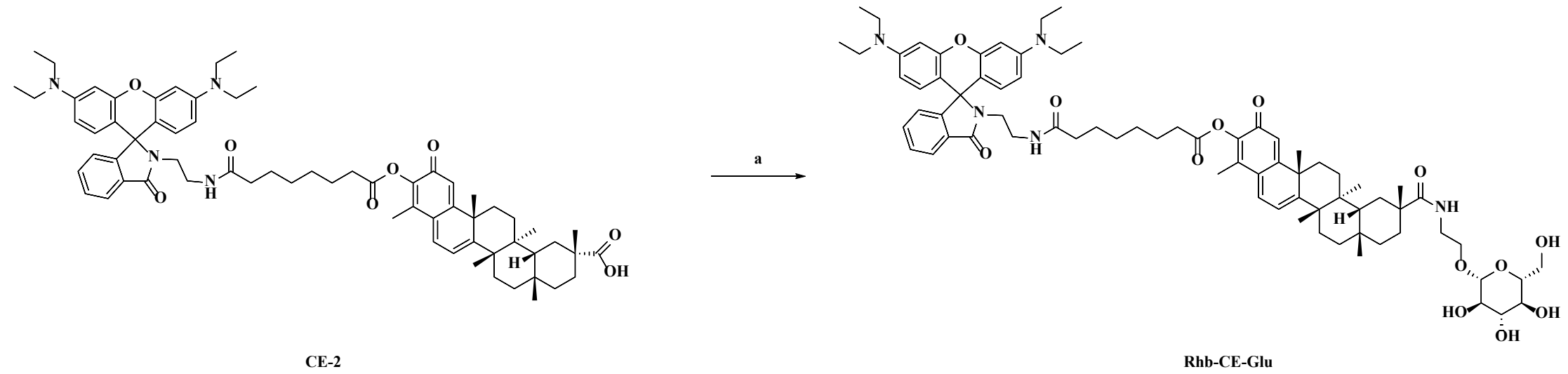

Scheme S4 Synthesis of Rhb-CE-Glu. (a) HATU, DIPEA, DMF, rt, 4 h.

3. ^{1}H NMR, ^{13}C NMR and HPLC analysis of synthesized compounds

A.

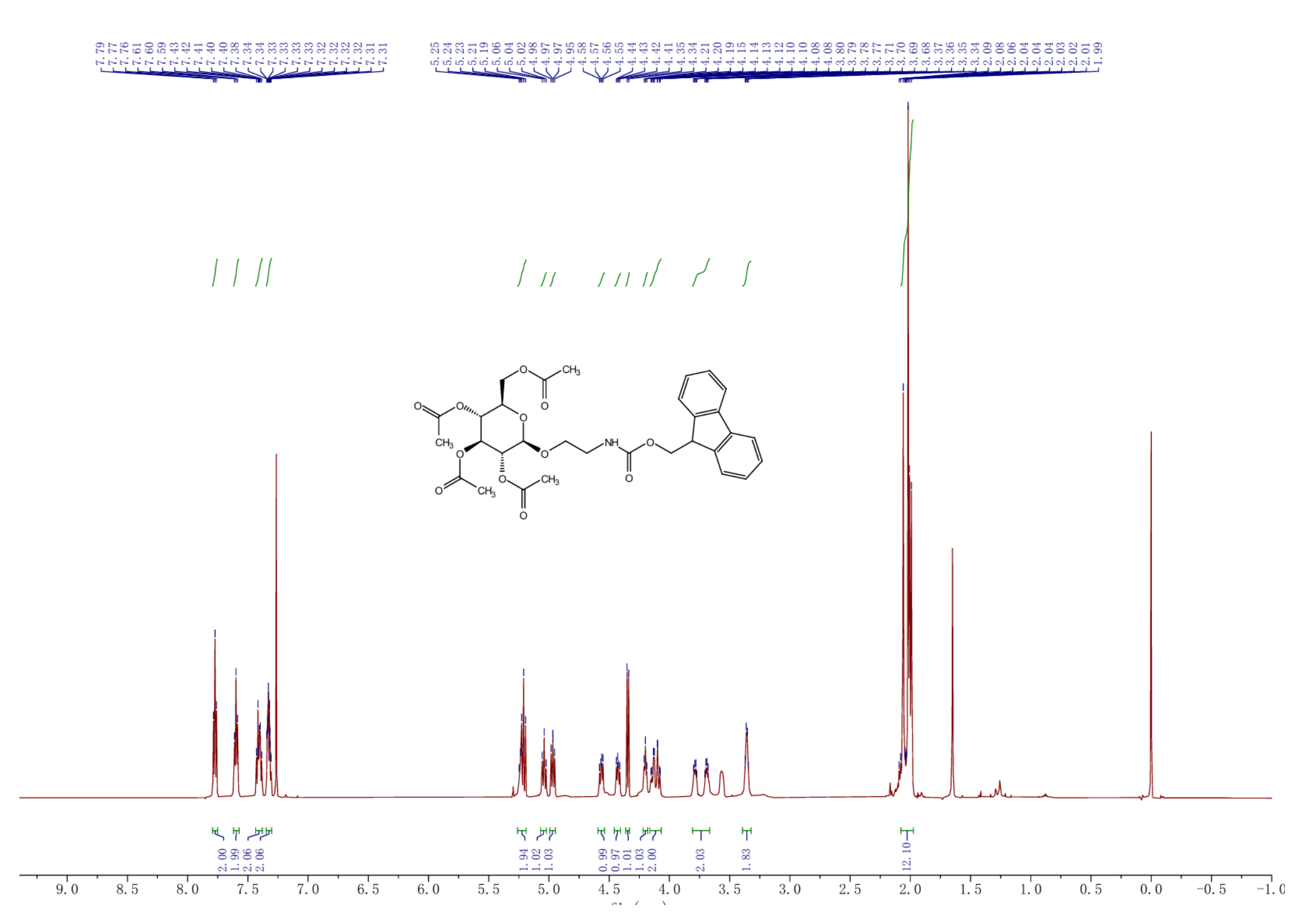

B.

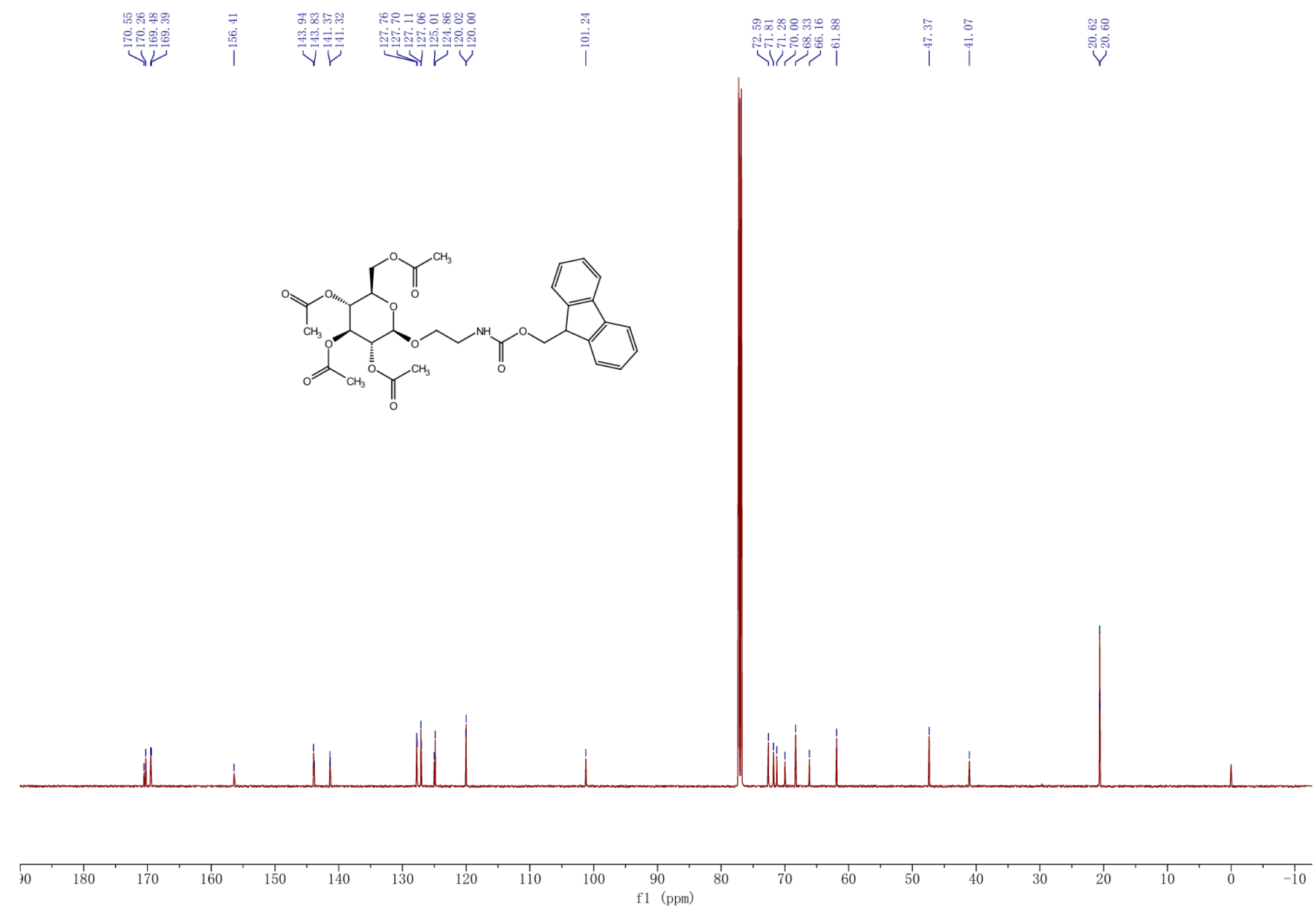

Figure S1 ^{1}H NMR (**A**) and ^{13}C NMR (**B**) spectrum of compound **2**.

A.

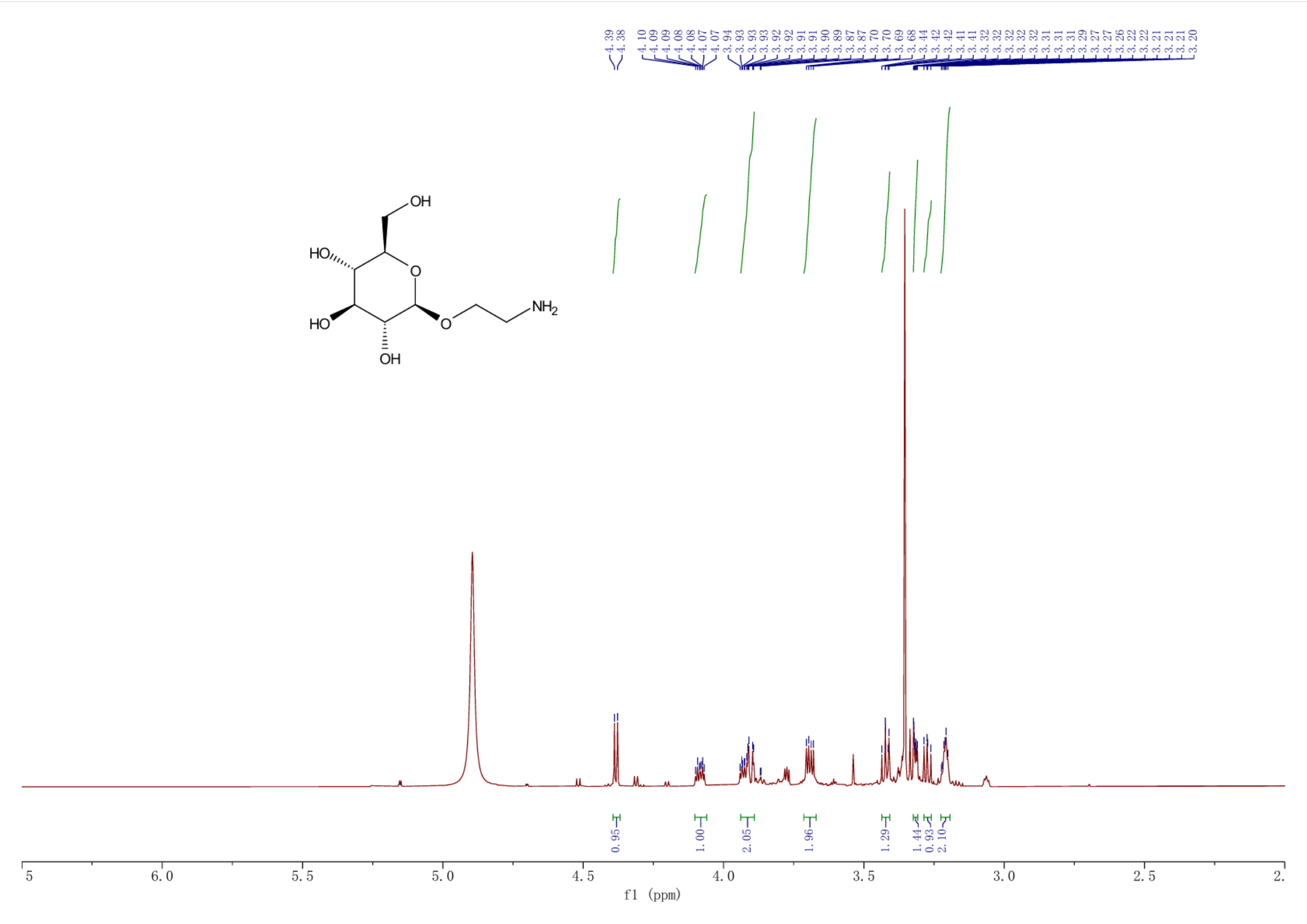

B.

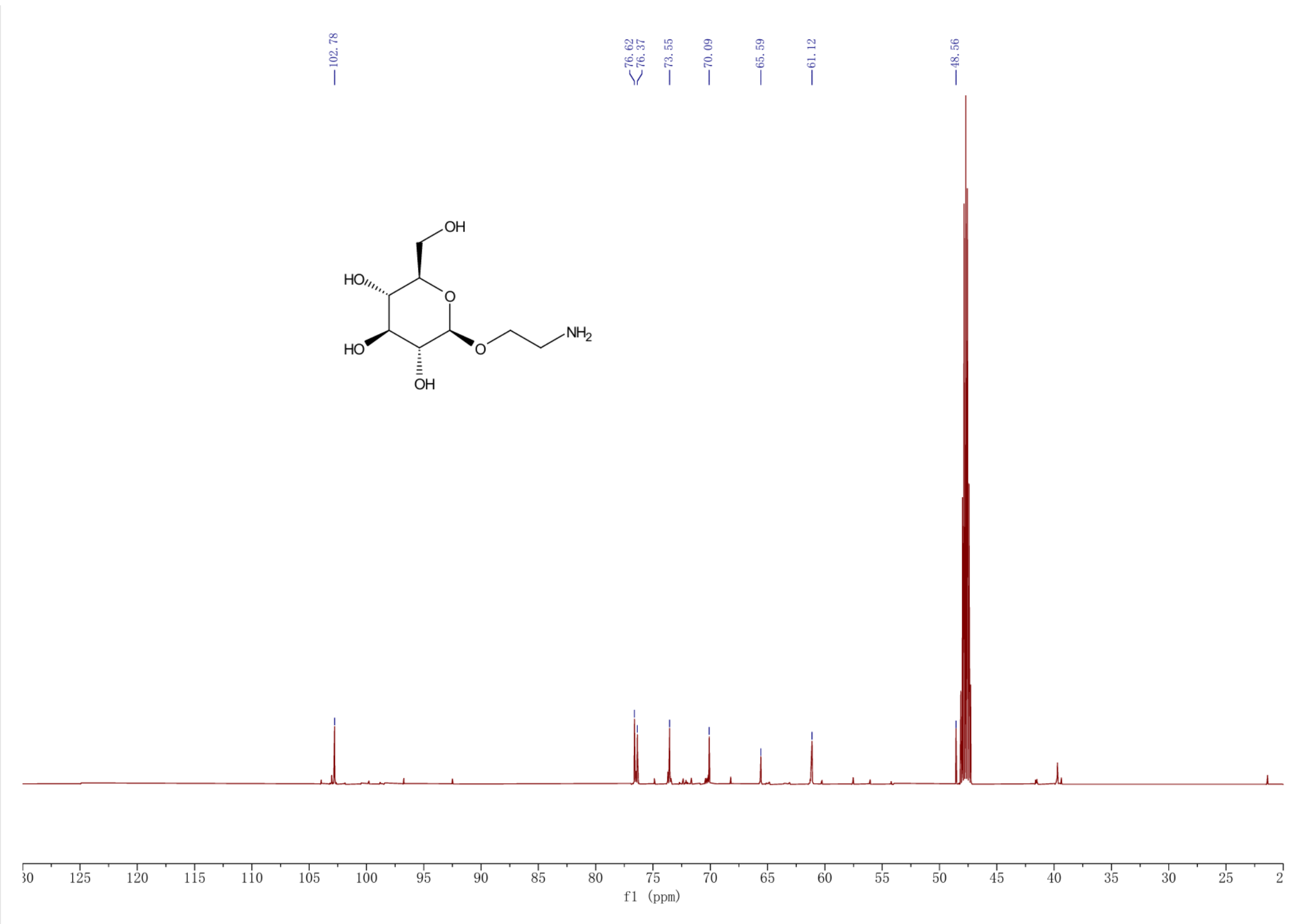

Figure S2 ^{1}H NMR (**A**) and ^{13}C NMR (**B**) spectrum of compound **3**.

A.

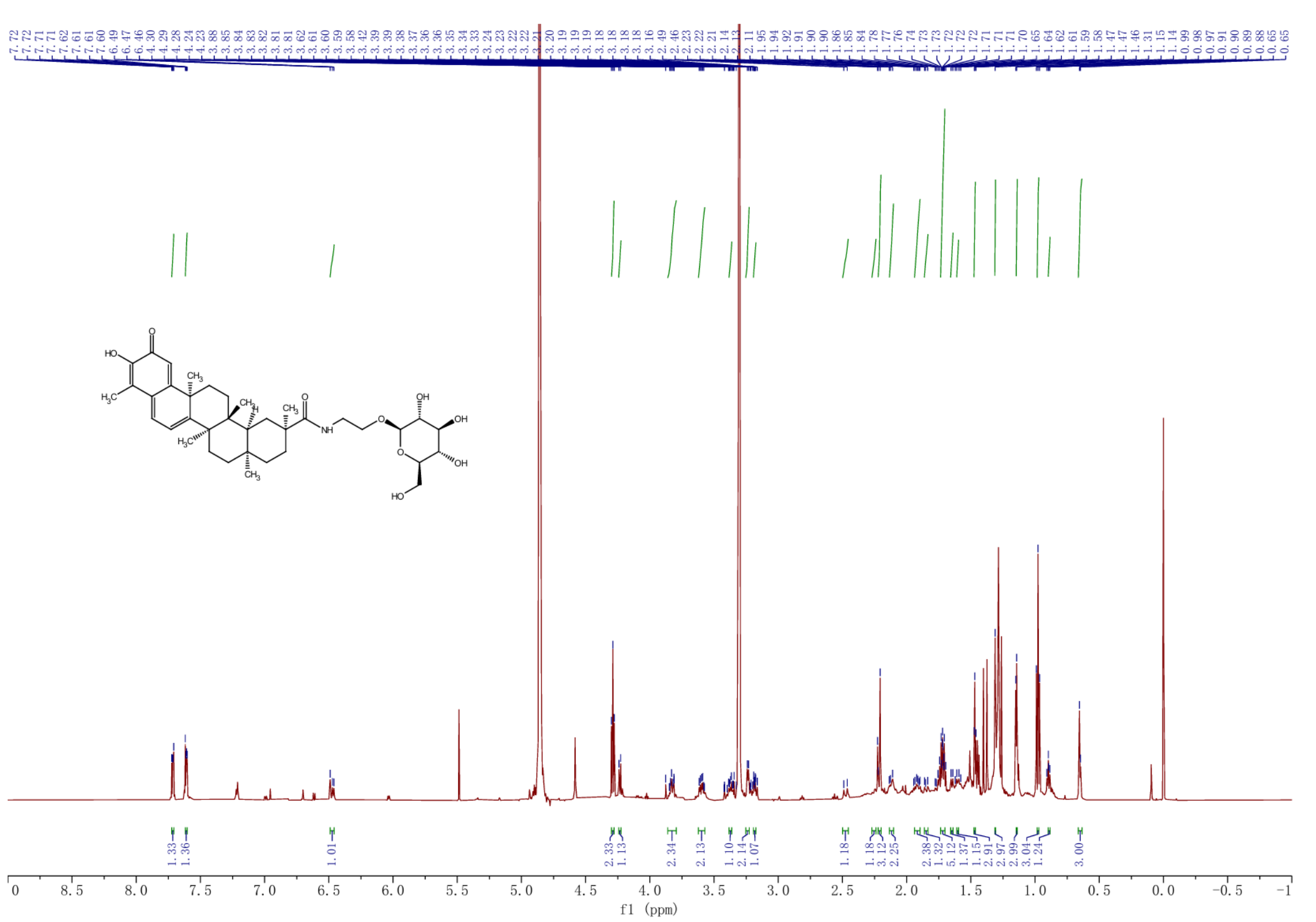

B.

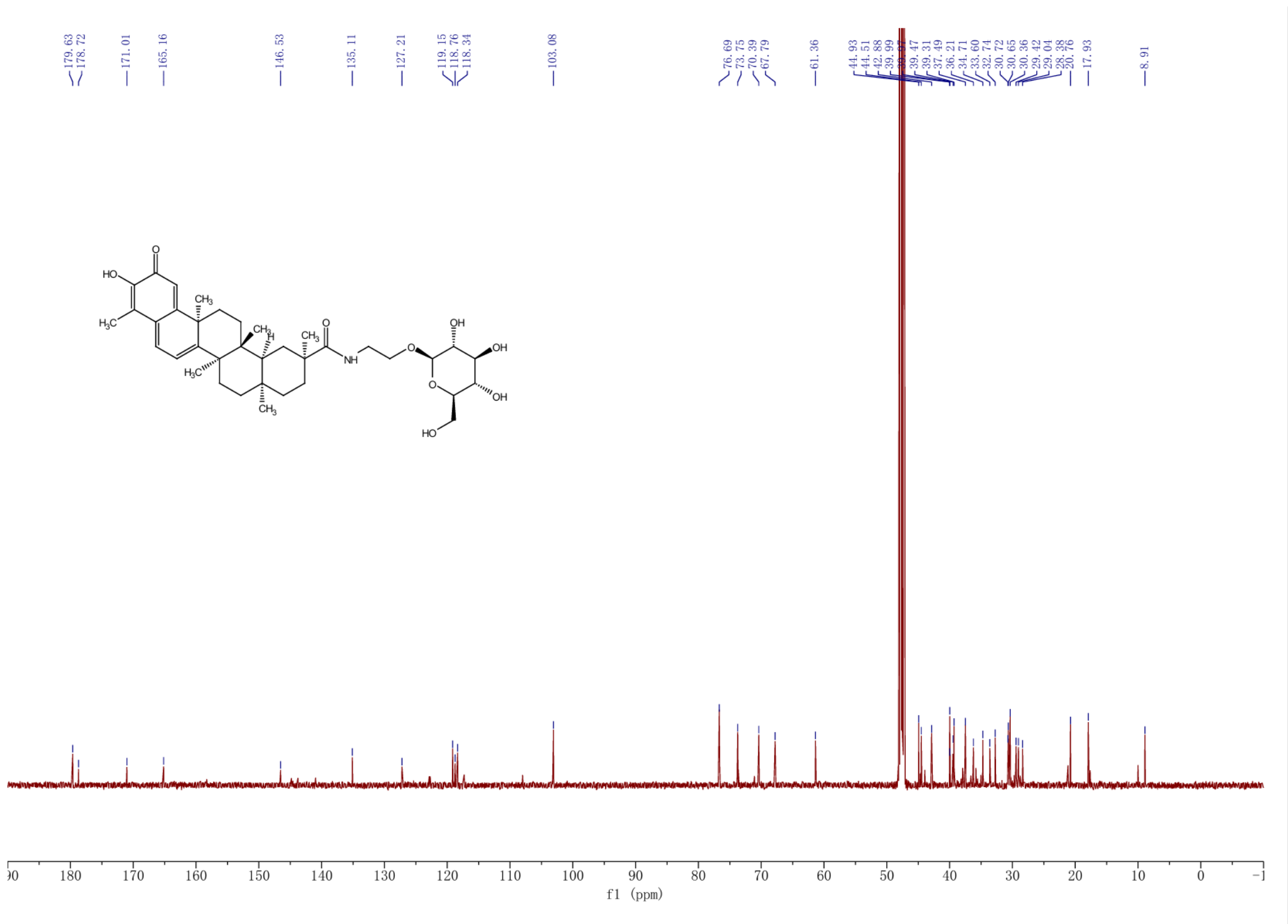

Figure S3 ^{1}H NMR (**A**) and ^{13}C NMR (**B**) spectrum of CG.

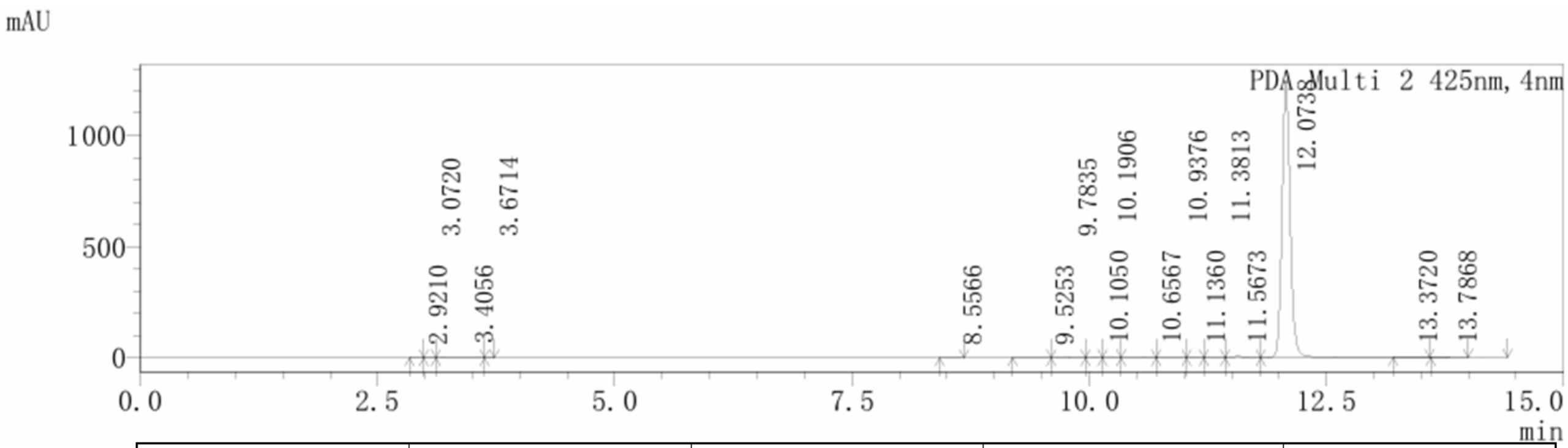

	RT	Area	Area %	Height
1	2.921	4046	0.053	633
2	3.072	4312	0.056	636
3	3.4056	31978	0.416	1885
4	3.6714	2073	0.027	502
5	8.5566	2901	0.038	600
6	9.5253	2799	0.036	251
7	9.7835	8365	0.109	732
8	10.105	6226	0.081	1098
9	10.1906	7642	0.099	1024
10	10.6567	10694	0.139	670
11	10.9376	13375	0.174	929
12	11.136	8203	0.107	812
13	11.3813	13463	0.175	1179
14	11.5673	52109	0.677	5484
15	12.0738	7479359	97.193	1253415
16	13.372	5024	0.065	701
17	13.7868	8342	0.108	1031
18	15.4186	2365	0.031	253
19	16.3601	8133	0.106	596
20	17.3952	17416	0.226	1485
21	20.1173	2418	0.031	173
22	20.434	4111	0.053	284

Figure S4 HPLC spectrum of CG (RT:12.0738 min, Purity:97.193%).

A.

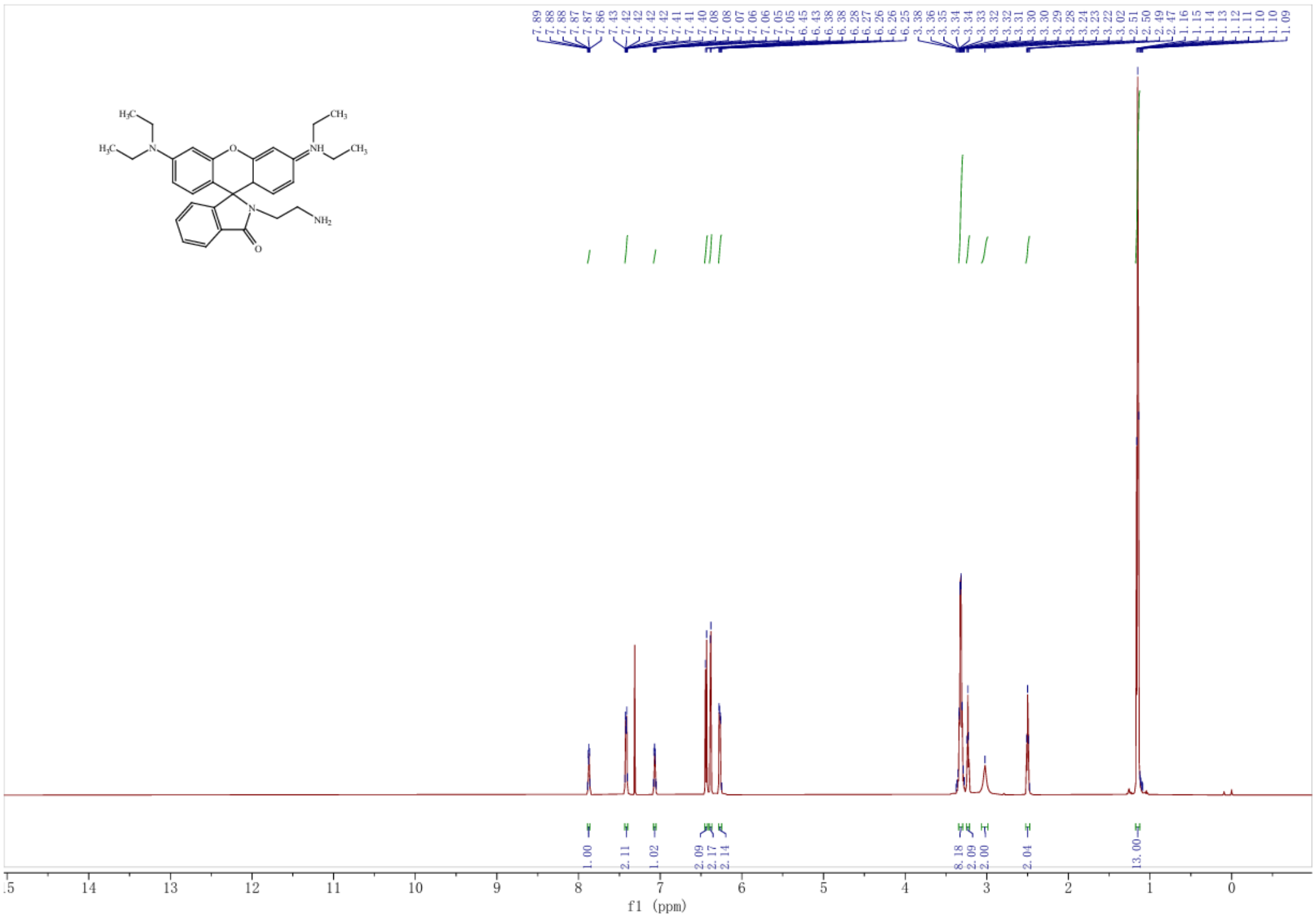

B.

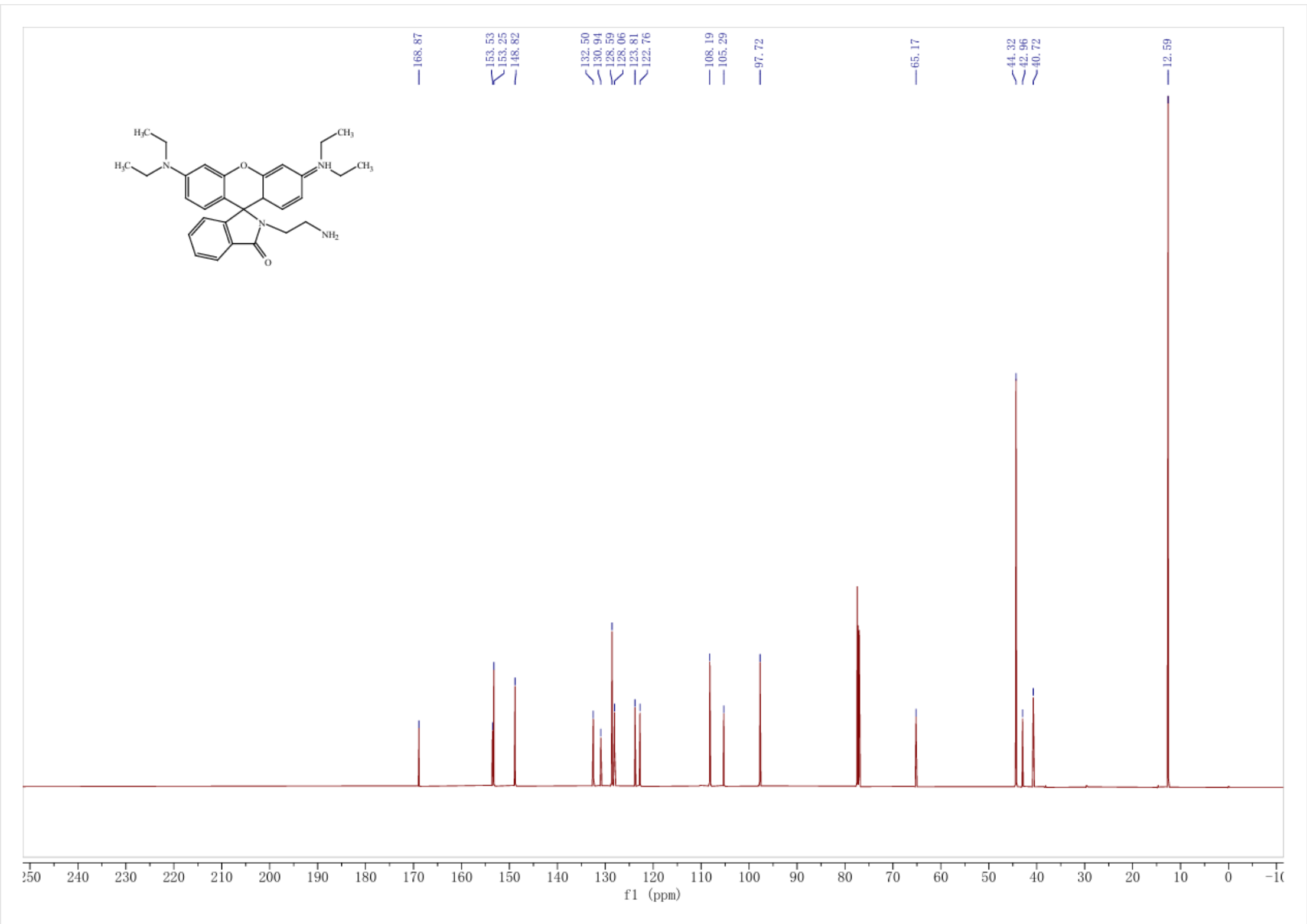

Figure S5 ^{1}H NMR (**A**) and ^{13}C NMR (**B**) spectrum of Rhodamine B-1.

A.

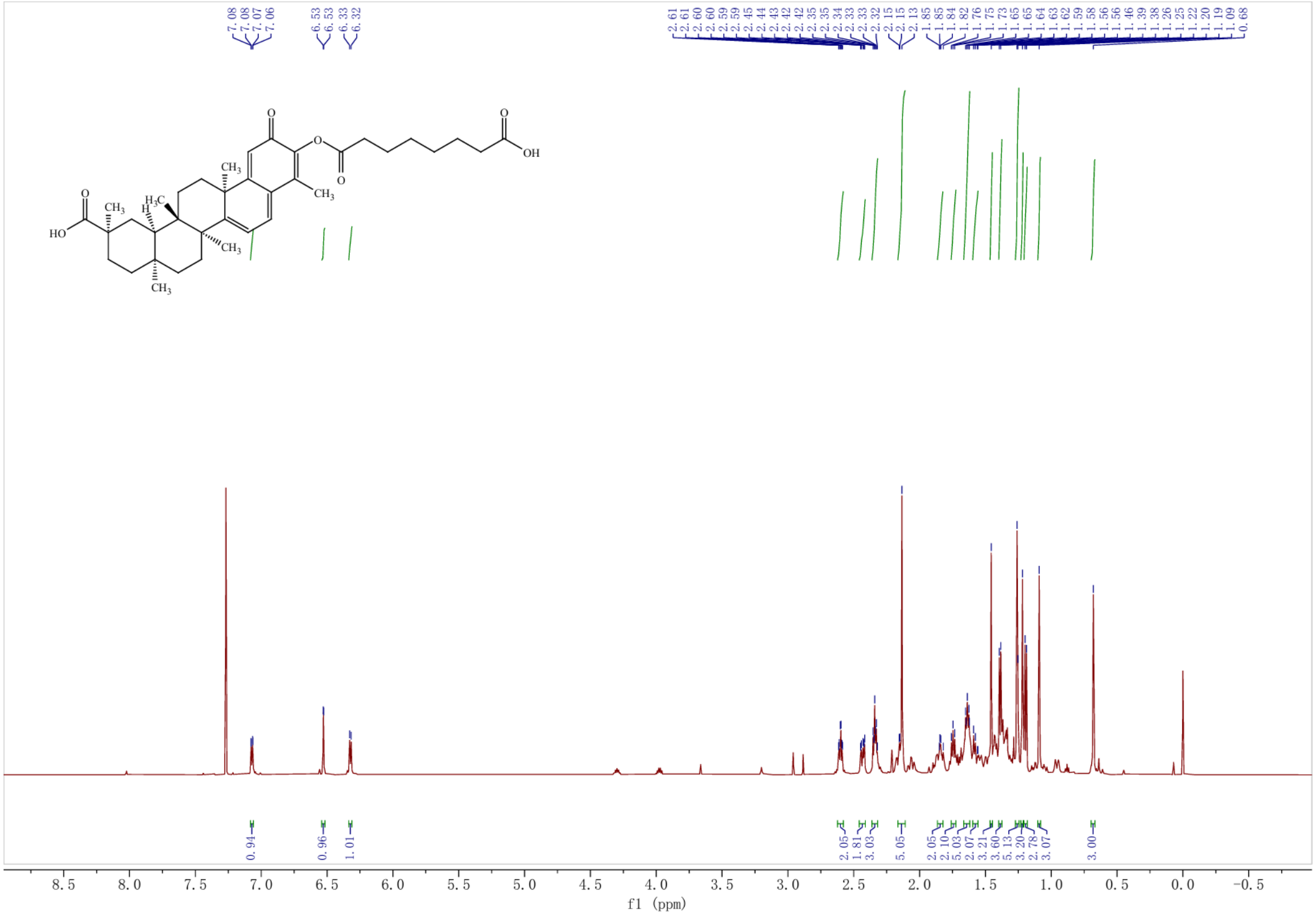

B.

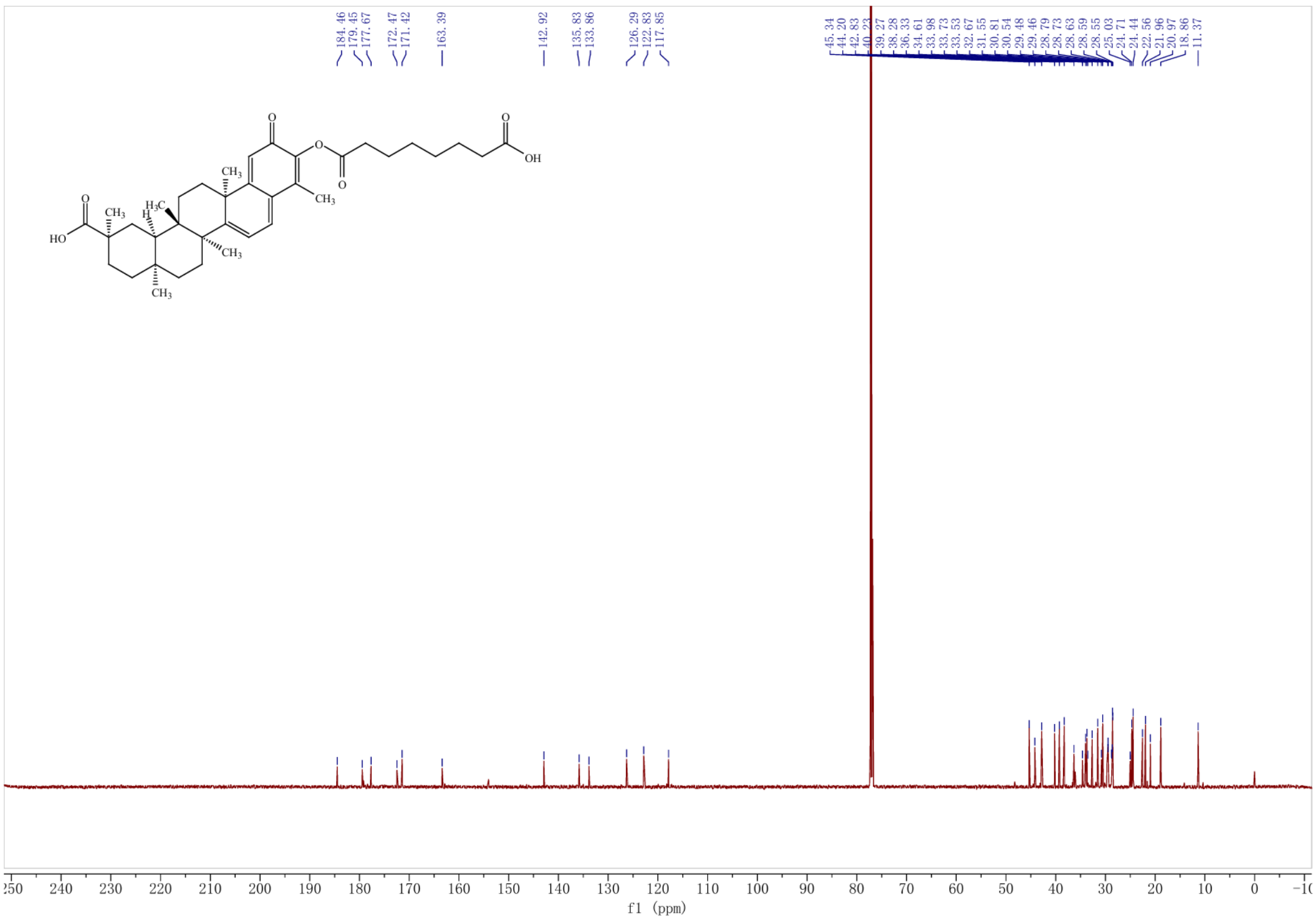

Figure S6 ^{1}H NMR (**A**) and ^{13}C NMR (**B**) spectrum of CE-1.

A.

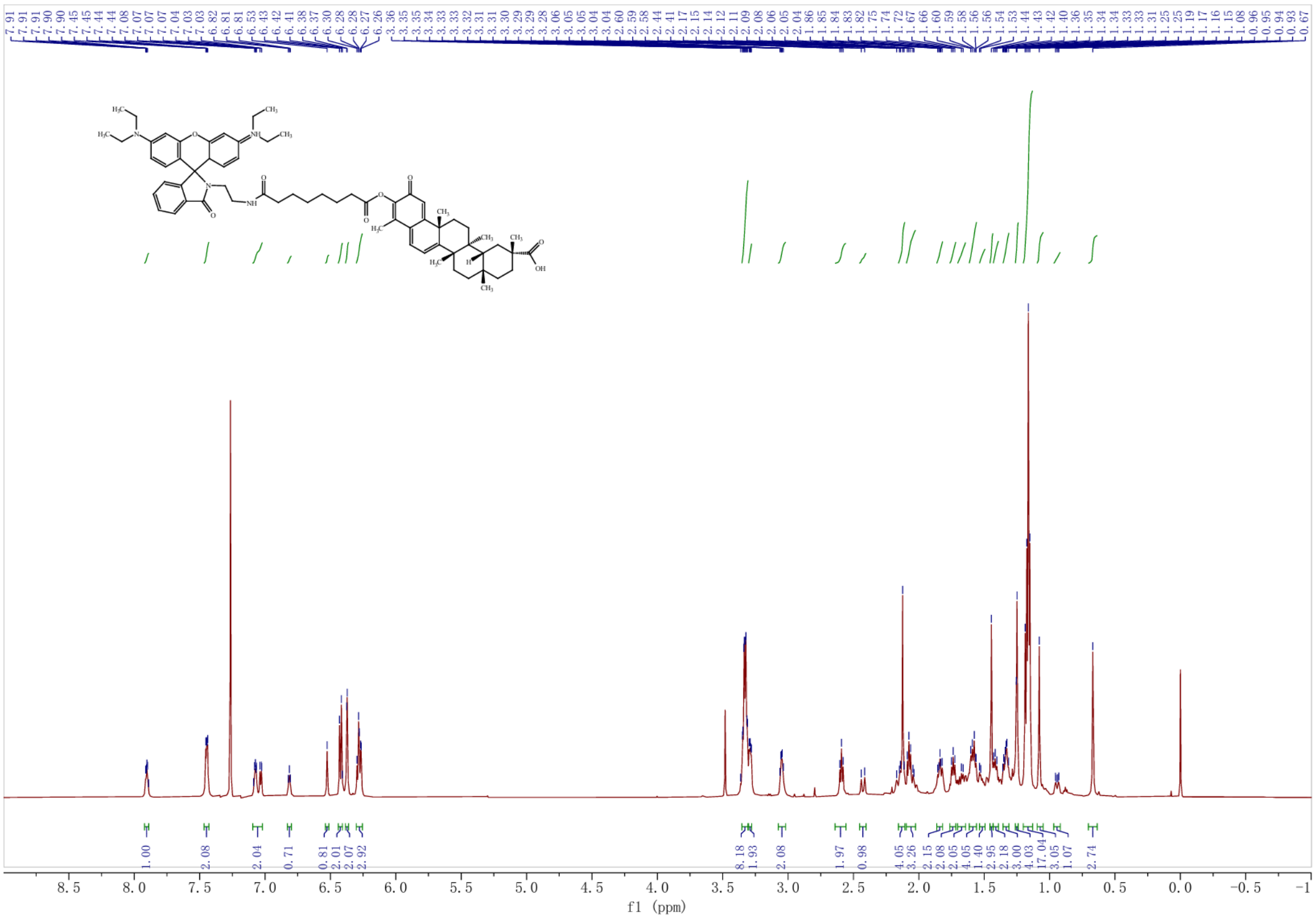

B.

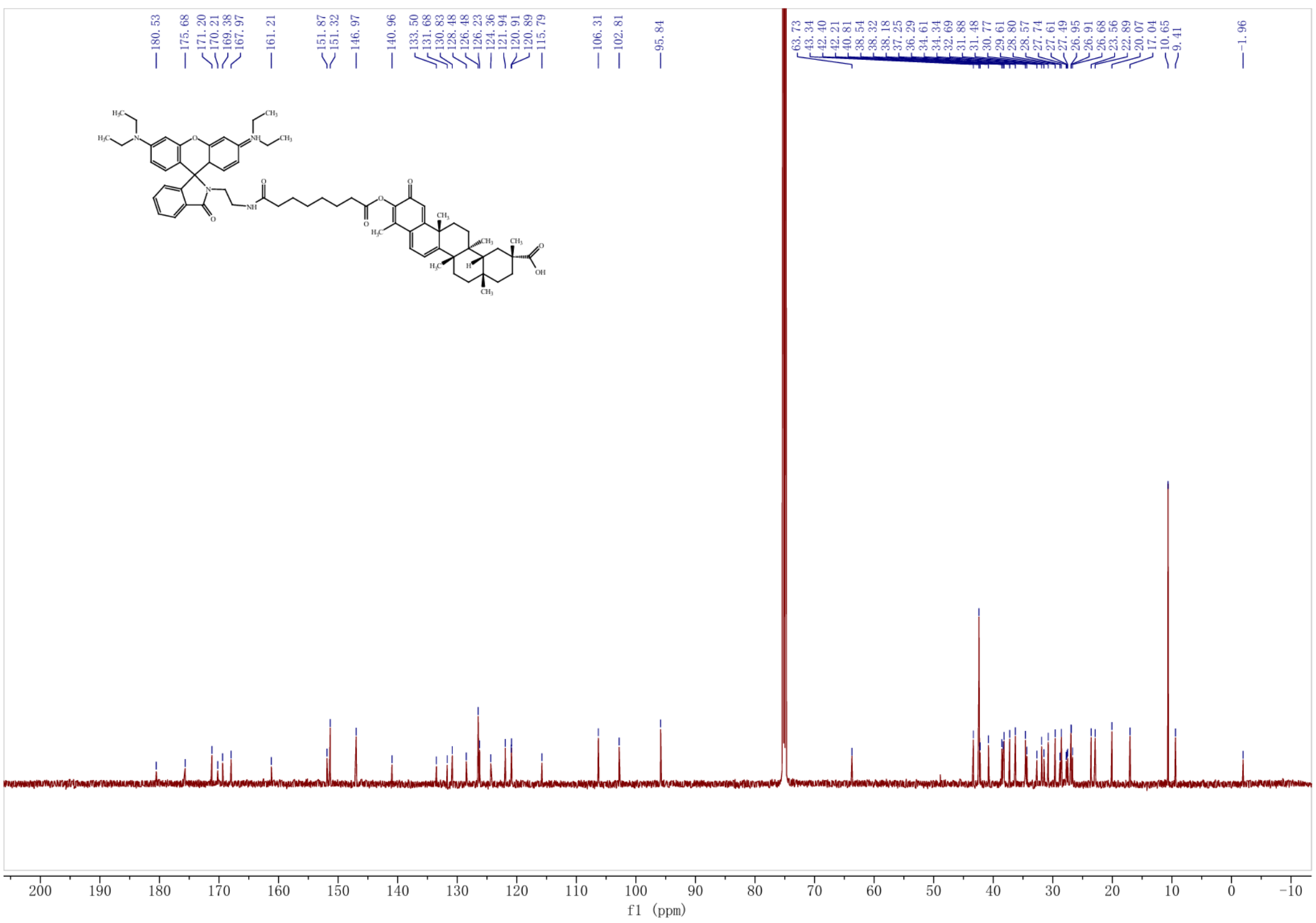

Figure S7 ^{1}H NMR (**A**) and ^{13}C NMR (**B**) spectrum of CE-2.

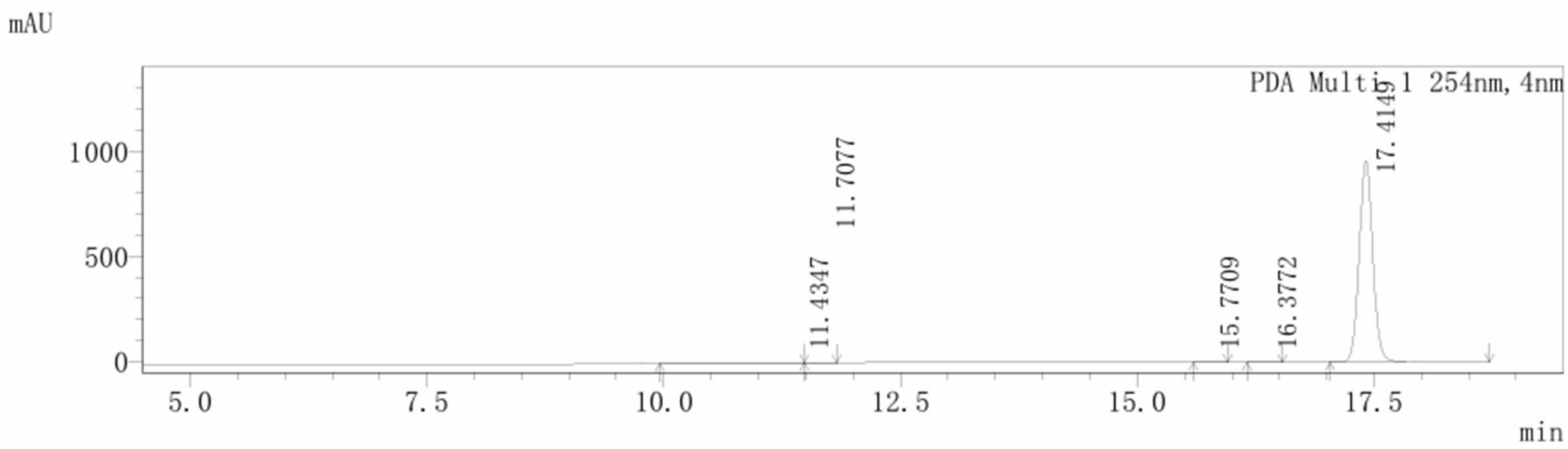

	RT	Area	Area %	Height
1	2.3724	98462	1.010	7524
2	2.6377	2183	0.022	649
3	2.8926	71822	0.737	5492
4	11.4347	47969	0.492	778
5	11.7077	7980	0.082	392
6	15.7709	2660	0.027	320
7	16.3772	1702	0.017	153
8	17.4149	9517233	97.613	954772

Figure S8 HPLC spectrum of CE-2 (RT:17.4149 min, Purity:97.613%).

A.

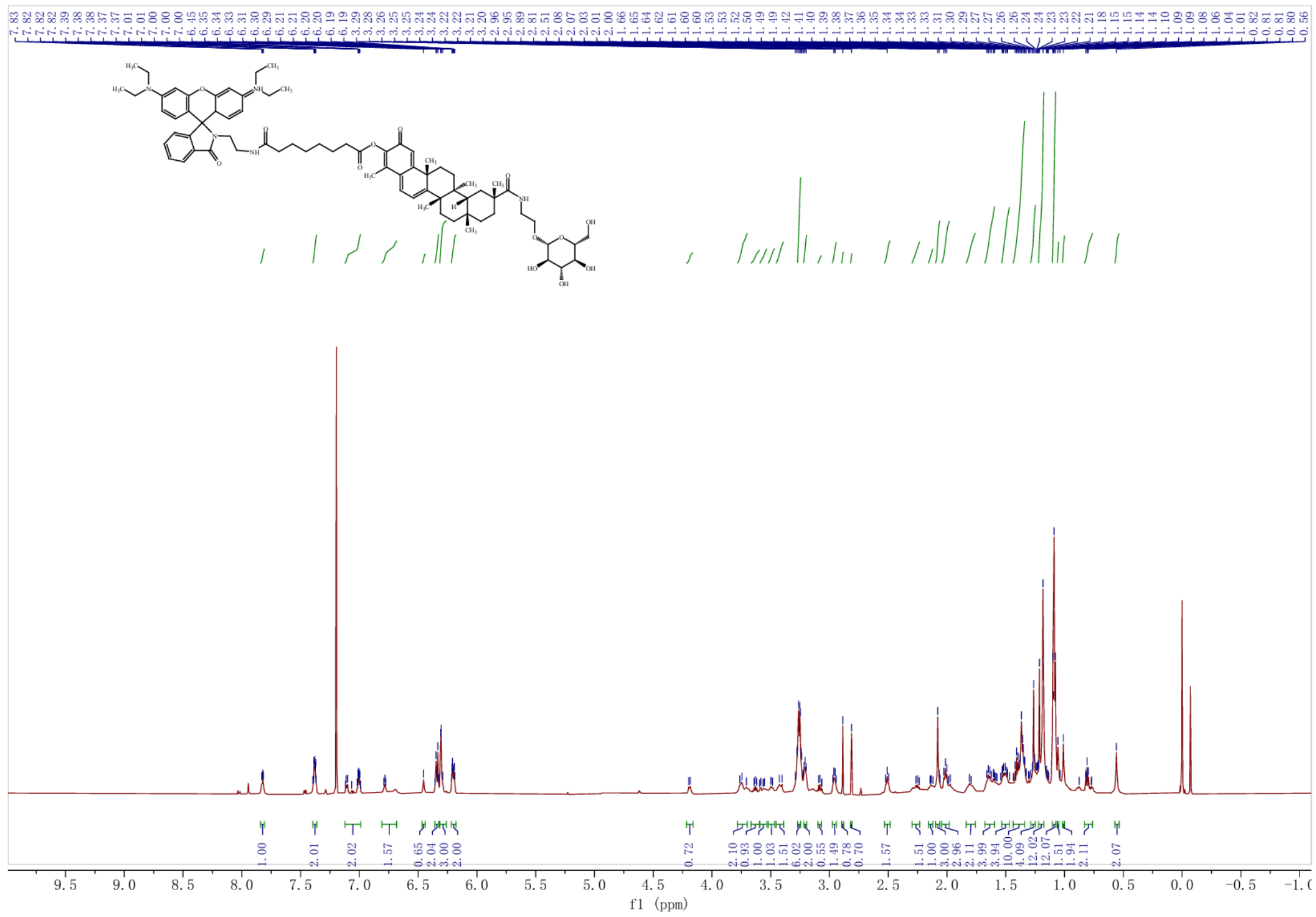

B.

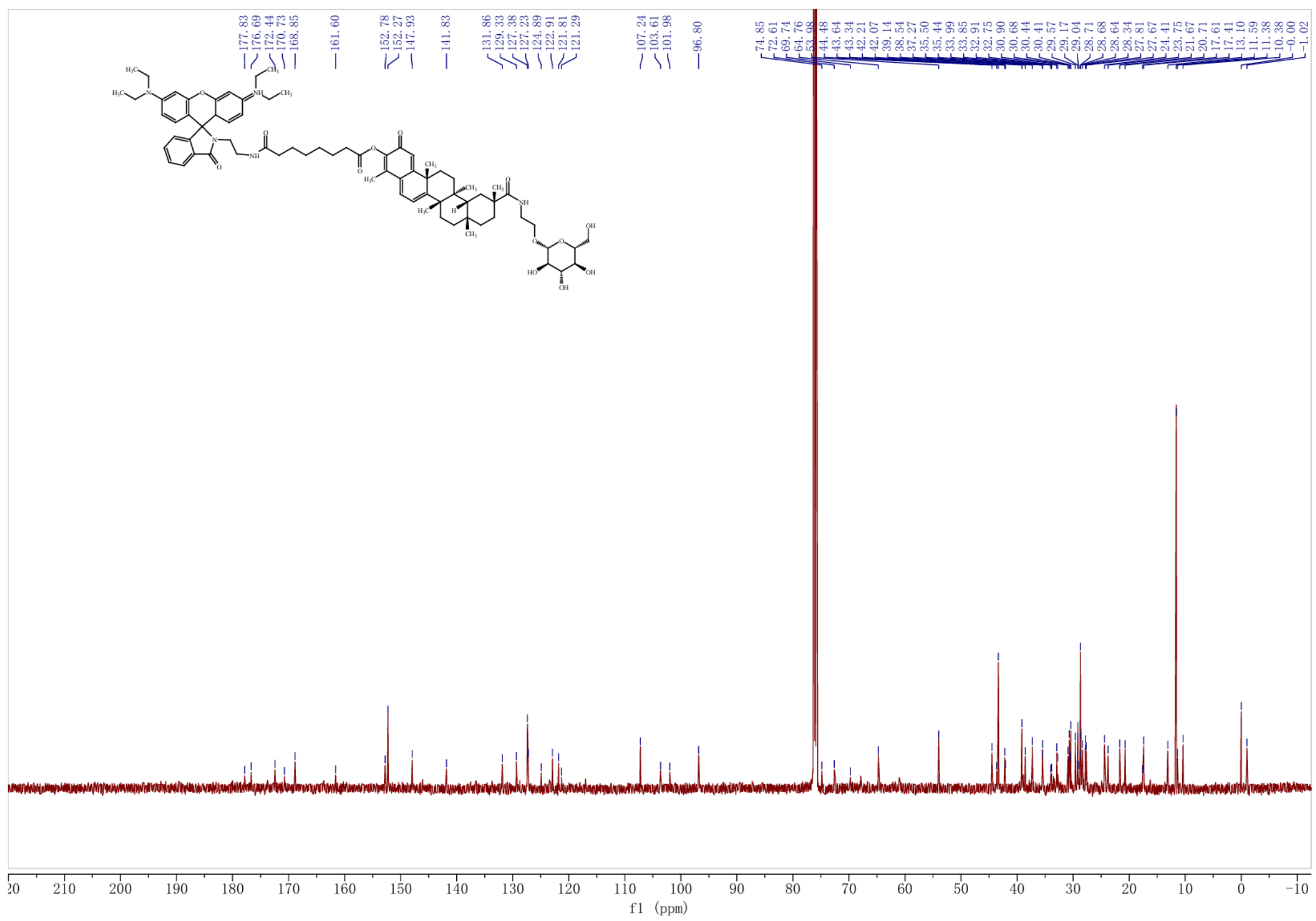

Figure S9 ^{1}H NMR (**A**) and ^{13}C NMR (**B**) spectrum of Rhb-CG.

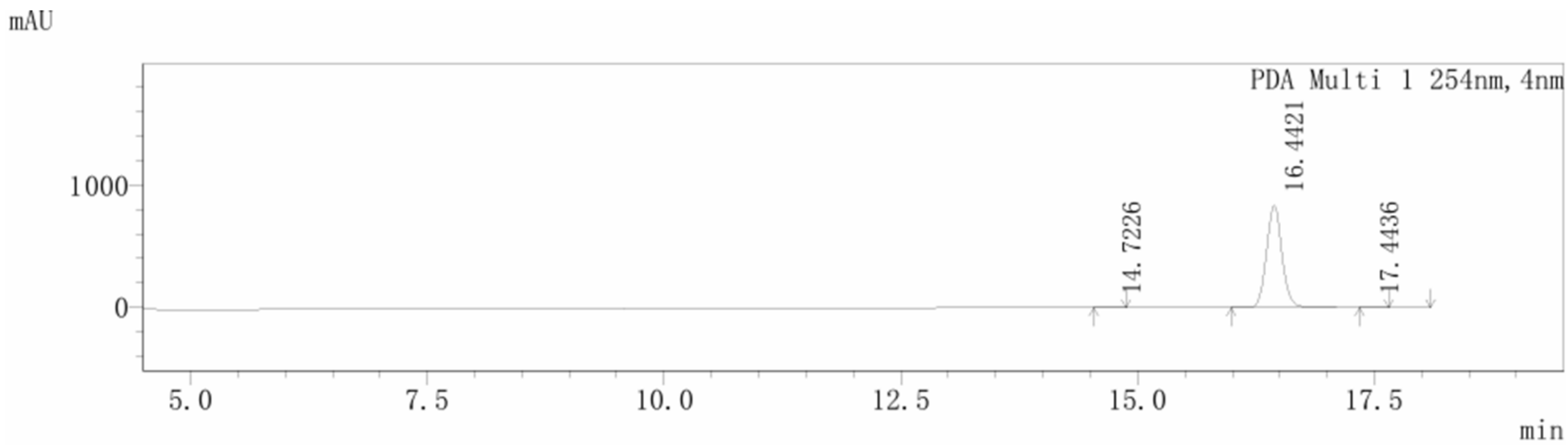

	RT	Area	Area %	Height
1	2.8347	9139	0.099	1888
2	3.1858	284596	3.079	6481
3	16.4421	8949096	96.82	817304

Figure S10 HPLC spectrum of Rhb-CG (RT:16.4421 min, Purity:96.82%).

4. Supplementary figures

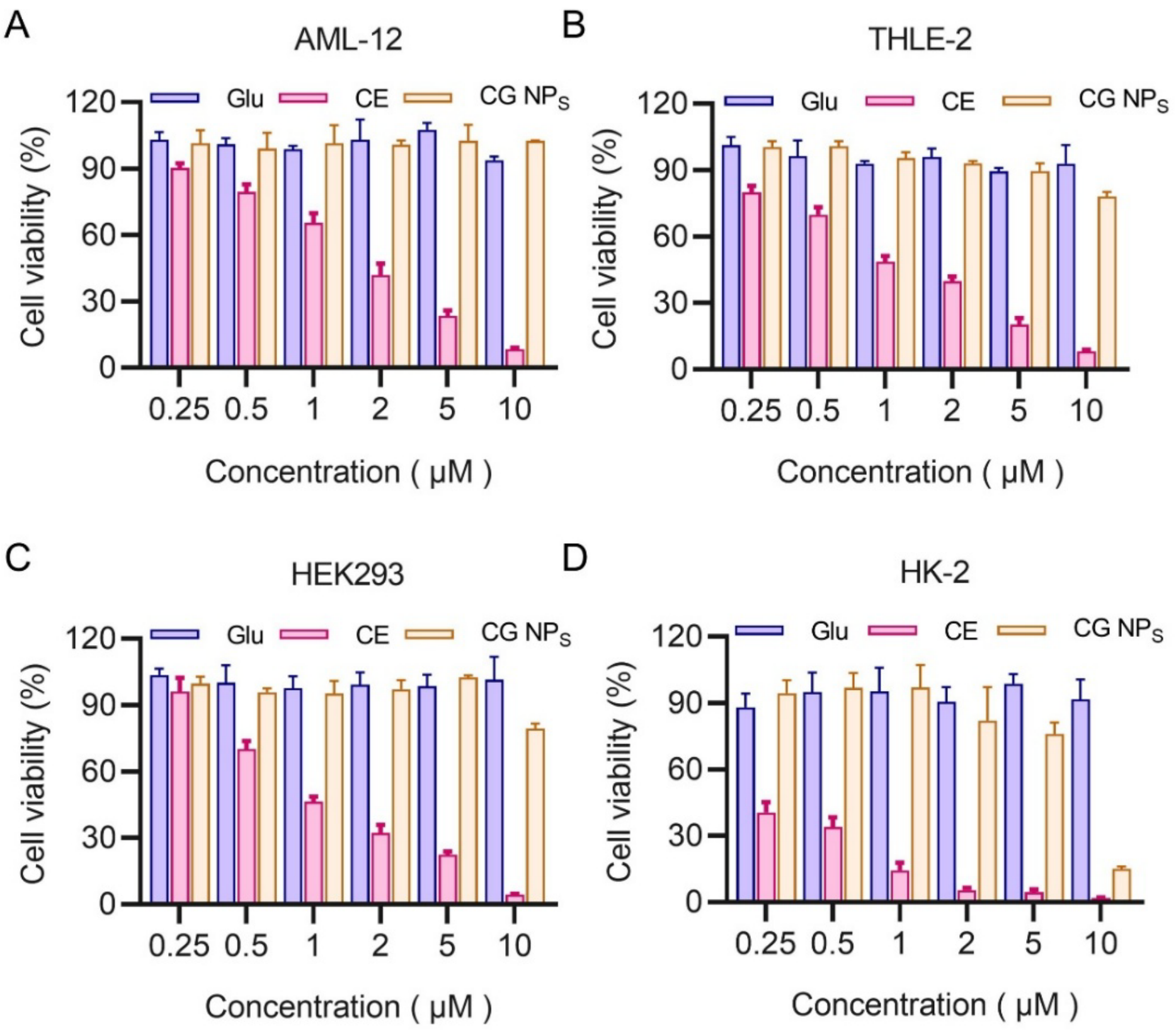

Figure S11 Cell viability of normal liver and kidney cells after drug treatment, as assessed by MTT assay.

Figure S12 (**A**) The inhibition rate of RAW264.7 cells treated with different drugs for 24 h. (**B**) The cytotoxicity assay conducted by lactate dehydrogenase (LDH) release.

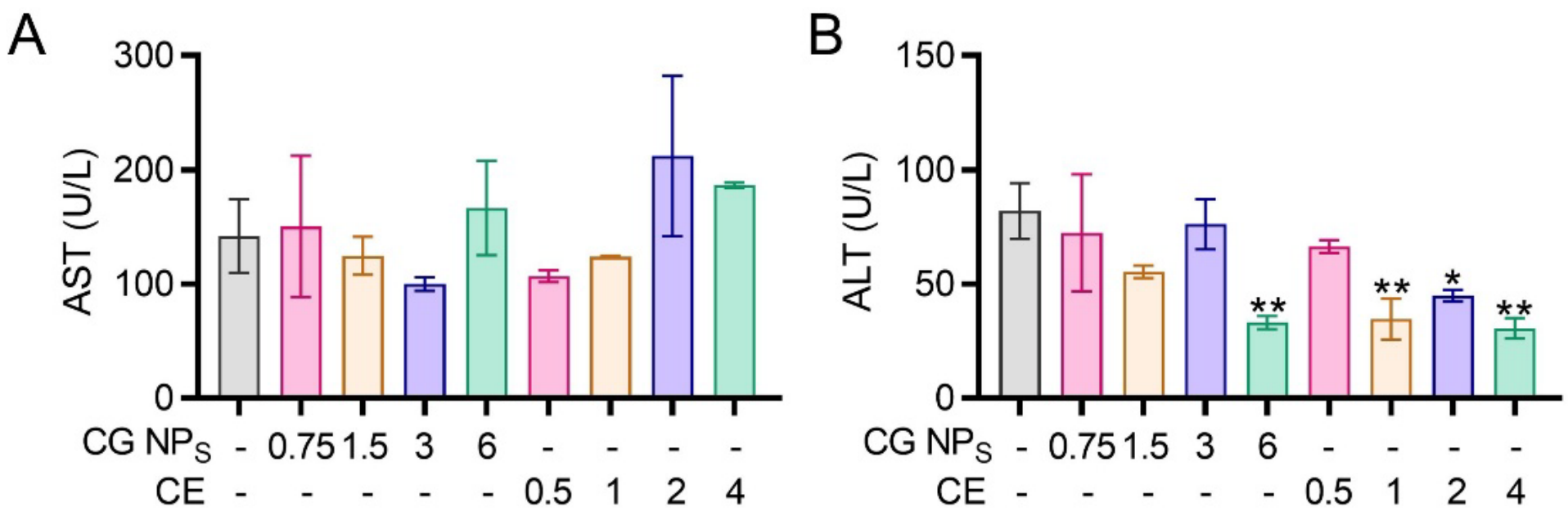

Figure S13 Hepatic biochemical profile post-acute administration. Serum concentrations of (**A**) aspartate aminotransferase (AST) and (**B**) alanine aminotransferase (ALT) in mice following acute treatment with CGNPs or CE.

Figure S14 (**A**) Flow cytometry histograms and (**B**) quantitative analysis of Rhb-CE and Rhb-CE-Glu uptake in Glu-pretreated M1 macrophages.

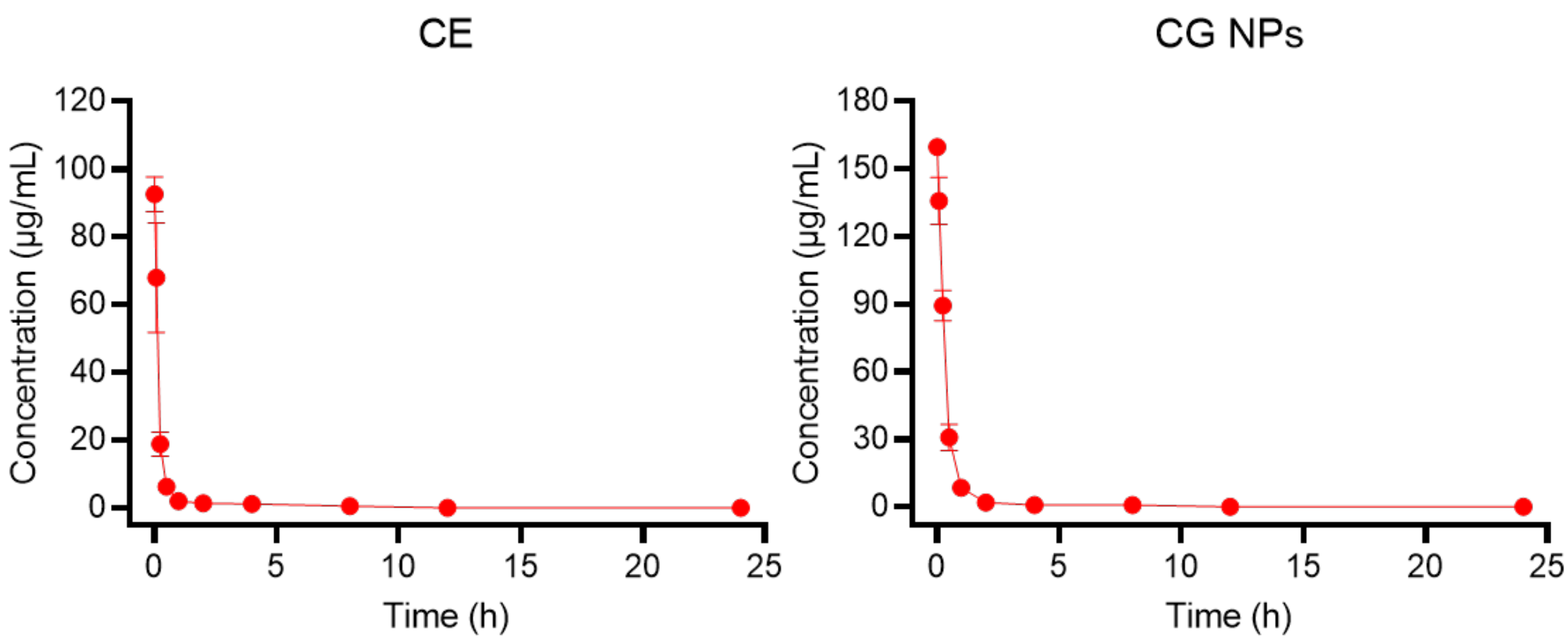

Figure S15 Plasma concentration-time curves of CE and CG NPs.

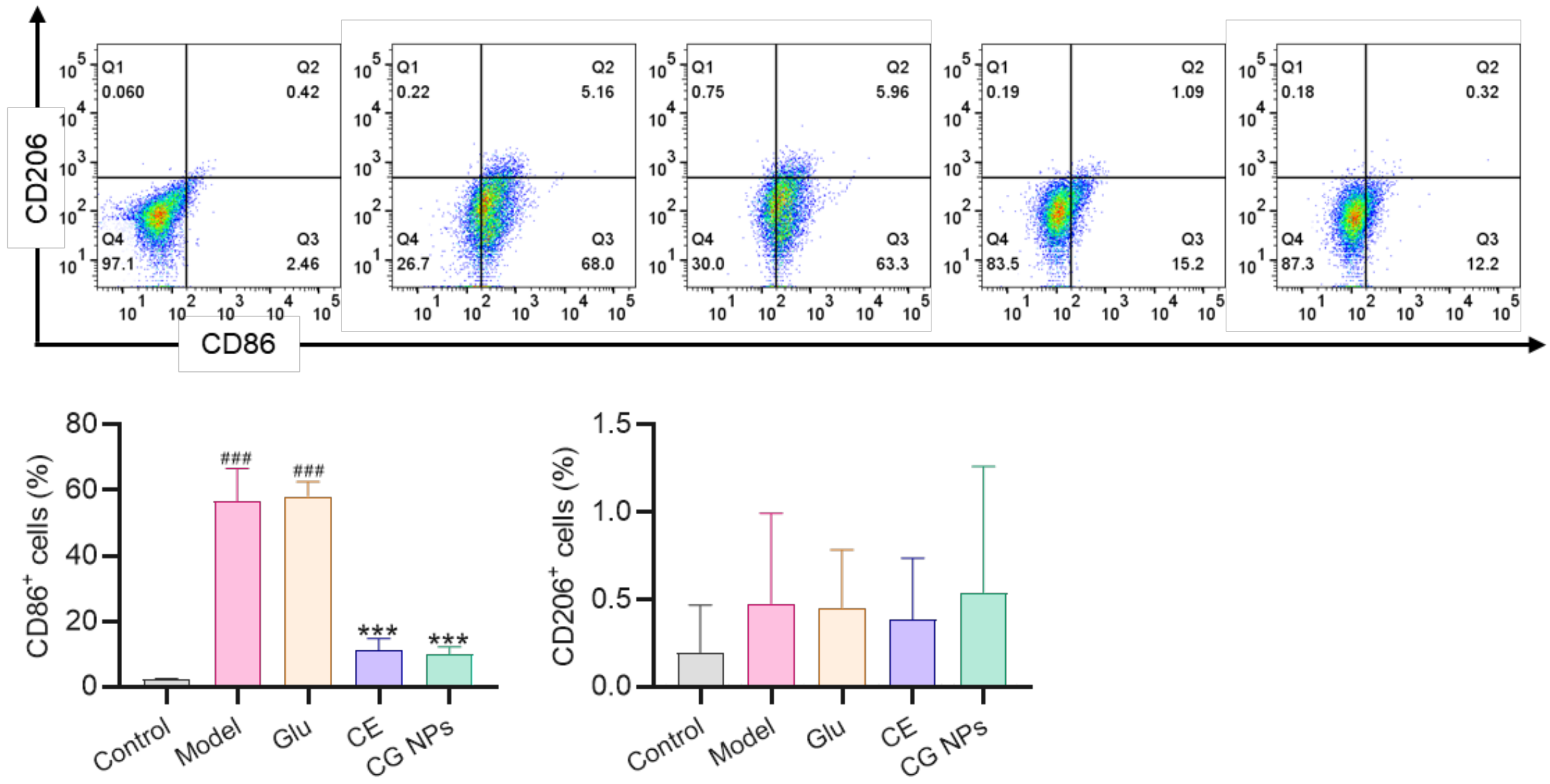

Figure S16. Representative plots of CD86 and CD206 expression and statistical analysis of the percentages of positive cells in macrophages from each group.

Table S1 Pharmacokinetic parameters of CE

Parameter	Unit	Value
$T_{1/2\alpha}$	h	0.134
$T_{1/2\beta}$	h	0.361
CL	L/h/kg	0.078
$AUC_{(0-t)}$	mg/L*h	23.745
$AUC_{(0-\infty)}$	mg/L*h	25.51
C_{max}	mg/L	90.674

Table S2. Pharmacokinetic parameters of CG NPs

Parameter	Unit	Value
$T_{1/2\alpha}$	h	0.259
$T_{1/2\beta}$	h	1.814
CL	L/h/kg	0.043
$AUC_{(0-t)}$	mg/L*h	67.438
$AUC_{(0-\infty)}$	mg/L*h	70.054
C_{max}	mg/L	157.796
